# The resistance to anoikis, mediated by Spp1, and the evasion of immune surveillance facilitate the invasion and metastasis of hepatocellular carcinoma

**DOI:** 10.1007/s10495-024-01994-x

**Published:** 2024-07-27

**Authors:** Zhengwei Zhang, Xiaoning Chen, Yapeng Li, Feng Zhang, Zhen Quan, Zhuo Wang, Yang Yang, Wei Si, Yuting Xiong, Jiaming Ju, Yu Bian, Shibo Sun

**Affiliations:** 1 The Second Affiliated Hospital of Harbin Medical University, Harbin, 150081 China; 2 Department of Pharmacology (National Key Laboratory of Frigid Zone Cardiovascular Diseases, Key Laboratory of Cardiovascular Research, Ministry of Education), College of Pharmacy, the State-Province Key Laboratories of Biomedicine-Pharmaceutics of China, Harbin Medical University, Harbin, 150081 China

**Keywords:** Hepatocellular carcinoma, Anoikis resistance, SPP1, Immune escape

## Abstract

Anoikis-Related Genes (ARGs) lead to the organism manifesting resistance to anoikis and are associated with unfavorable prognostic outcomes across various malignancies.Therefore, it is crucial to identify the pivotal target genes related to anoikis in HCC .We found that ARGs were significantly correlated with prognosis and immune responses in HCC. The core gene, SPP1, notably promoted anoikis resistance and metastasis in HCC through both in vivo and in vitro studies. The PI3K-Akt-mTOR pathway played a critical role in anoikis suppression within HCC contexts. Our research unveiled SPP1’s role in enhancing PKCα phosphorylation, which in turn activated the PI3K-Akt-mTOR cascade. Additionally, SPP1 was identified as a key regulator of MDSCs and Tregs migration, directly affecting their immunosuppressive capabilities.These findings indicate that in HCC, SPP1 promoted anoikis resistance and facilitated immune evasion by modulating MDSCs and Tregs.

**Graphical Abstract:**

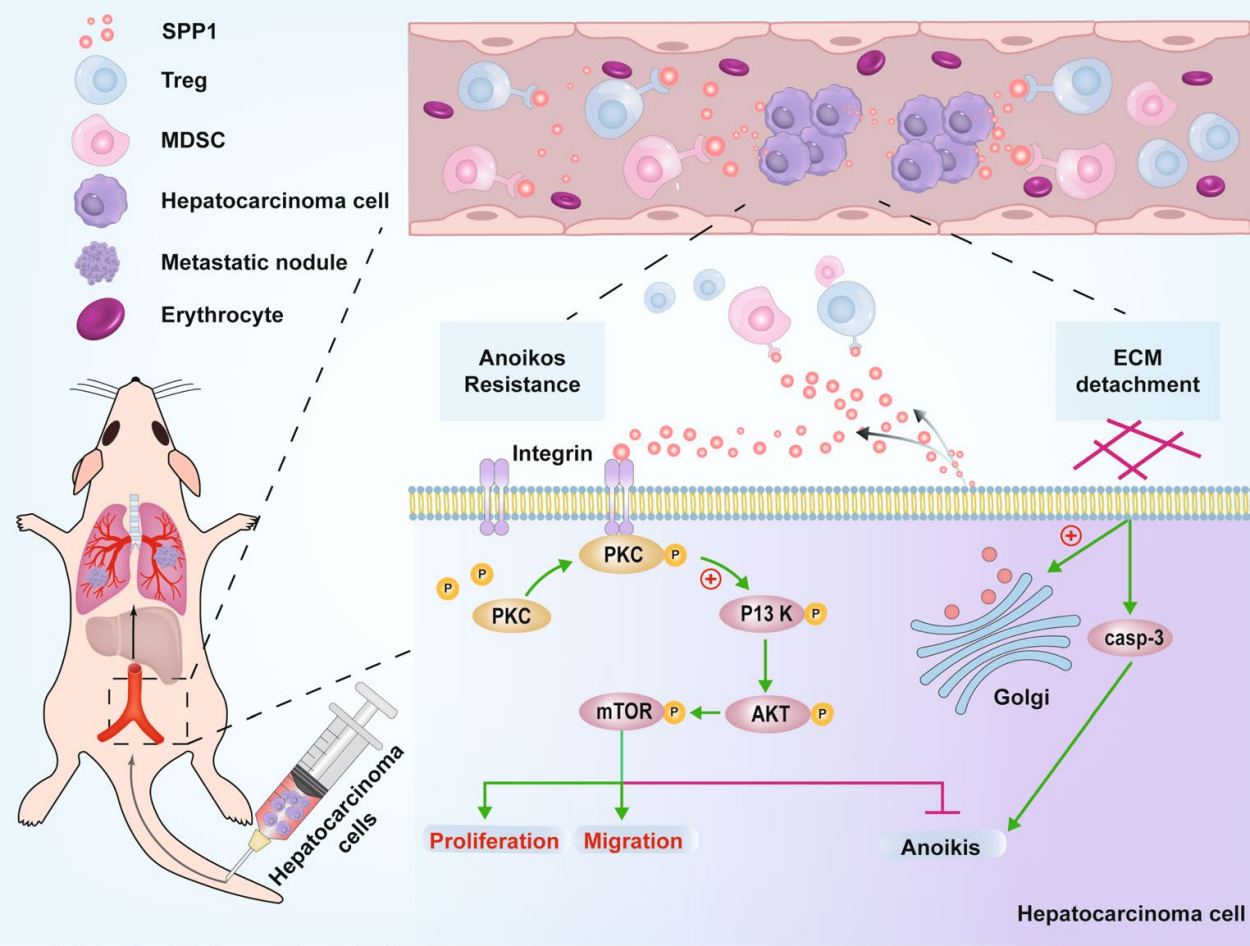

**Supplementary Information:**

The online version contains supplementary material available at 10.1007/s10495-024-01994-x.

## Introduction

Precisely, HCC is ranked as the sixth most prevalent cancer globally and the third leading cause of cancer-related mortality [1]. It represents the primary type of liver cancer [2]. While surgical intervention offers a potential cure, the aggressive and elusive nature of HCC often excludes many patients from surgical resection [3]. Additionally, postoperative recurrence and metastasis are common in clinical settings, significantly influencing the prognosis of HCC patients adversely [4]. Metastasis is crucial in tumor progression and represents the primary obstacle to improving long-term survival rates for patients. The metastatic process is complex, involving the detachment of cancer cells from the primary tumor, invasion into neighboring tissues, intravasation into the bloodstream or lymphatic system, survival during transit, and establishment of secondary tumors at distant sites [5]. The evolution of resistance against anoikis is considered an essential prerequisite for initiating and fostering metastasis [6]. Anoikis specifically refers to programmed cell death triggered by the absence of cellular engagement with the extracellular matrix or neighboring cells, which activates apoptosis through established pathways. Cancer cells that acquire resistance against anoikis are capable of surviving and proliferating independently without anchorage, facilitating the formation of distant metastases [7, 8]. Therefore, mastering such resilience becomes vital for liver cancer’s intrahepatic and extrahepatic dissemination. Exploring the molecular mechanisms underlying this phenomenon would substantially contribute to improving liver cancer treatment outcomes. However, currently, only a limited number of studies have employed bioinformatics methodologies to investigate the corresponding molecular mechanisms, resulting in an exceedingly restricted amount of elucidation and experimental validation. Consequently, the exact molecular processes accountable for the anti-anoikis effects in liver cancer remain obscure.

The intricate molecular mechanisms promoting metastasis also encompass an immune microenvironment that permits or sustains dissemination [6]. Even if circulating tumor cells successfully detach from the primary tumor, they still encounter immune surveillance within the bloodstream. Despite the identification of intimate associations between circulating tumor cells and various immune cells, their mechanism for evading immunity remains elusive [9]. Investigating the interaction mechanisms between circulating tumor cells and immune cells can lead to a more comprehensive understanding of cancer metastasis in patients and offer efficacious treatment strategies.

The current study utilized a comprehensive bioinformatics approach to identify ARGs that influence the prognosis of HCC. We performed unsupervised clustering analysis on prognostic ARGs, using diverse data sets, and followed this with enrichment analysis to investigate potential biological pathways. To identify the most effective prognostic risk model, we developed a panel of eighty machine learning algorithms. Additionally, we examined the relationship between immune response and ARGs. Notably, SPP1 was identified as a key target gene, and thorough experimental validation underscored its role in fostering resistance to anoikis in HCC by modulating the PI3K/AKT/mTOR signaling pathway. Significantly, we highlighted the function of SPP1 in recruiting immunosuppressive cells, such as MDSCs and Tregs. These insights suggested that SPP1 could be a valuable therapeutic target in the treatment of metastatic HCC.

## Materials and methods

### Bioinformatics processing and analysis

The gene expression profiles of HCC were meticulously extracted from The Cancer Genome Atlas (TCGA) and Gene Expression Omnibus (GEO) databases. Stringent inclusion criteria were applied, which involved the exclusion of samples with incomplete follow-up information, absence of survival days data, and repeated sequencing in the same patient. As a result, a total of 365 tumor samples from the TCGA-Liver Hepatocellular Carcinoma (LIHC) cohort and 221 tumor samples from the GSE14520 cohort were included. Additionally, an external validation cohort consisting of 231 HCC patients from the International Cancer Genome Consortium (ICGC)-LIHC cohort was incorporated into the machine learning model following identical inclusion and exclusion criteria. Somatic mutation data and copy number variation (CNV) data were meticulously acquired from the TCGA-LIHC cohort encompassing 371 tumor samples. The Maftools package was utilized to present mutation profiles while the sva package mitigated batch effects between RNA-seq and microarray data sources. A comprehensive inventory comprising 434 ARGs, extracted from both previous literature and GeneCards [10], was employed (Supplementary Table 1).

In our molecular subtyping pipeline, we designated the GSE14520 and TCGA-LIHC datasets as meta-cohorts to evaluate the prognostic value of individual ARGs through single-factor Cox regression analysis. Both TCGA and GEO cohorts underwent unsupervised consensus clustering analysis based on prognostic genes, followed by Principal Component Analysis (PCA) to determine the relative independence of each subtype. We utilized the R package ‘consensusClusterPlus’ with 100 repetitions and a pltem threshold of 0.8 to facilitate determining cluster numbers and validating subtype stability. Kaplan-Meier curves were used to assess overall survival (OS) in various LIHC patients within the dataset, supplemented by log-rank tests.

The limma package was used to conduct differential gene analysis within the TCGA cohort, aiming to identify significant genes across different subtypes (*adj.p* < 0.05, |logFC| > 1). Subsequently, the clusterProfiler package was utilized for gene ontology (GO) and Kyoto Encyclopedia of Genes and Genomes (KEGG) pathway annotation. Pathways with a *p* value < 0.05 and q-value < 0.05 were considered significantly enriched. Furthermore, GSVA was implemented to assess biological pathway differences among subtypes using c2.cp.kegg.v7.0.symbols.gmt as the reference gene set, with an FDR threshold of < 0.05.

To construct a prognostic model for HCC patients, we strictly adhered to previously established methodological framework [11, 12]. In the machine learning paradigm, we leveraged the TCGA-LIHC cohort for model development, further incorporating data from the GSE14520 and ICGC-LIHC cohorts for validation. Initially, ARGs were extracted separately from each cohort, and common genes across all cohorts were identified. Subsequently, data normalization was carried out within each cohort by standardizing gene mean/standard deviation to 1 with a minimum threshold of 4 variables for selection. In the TCGA-LIHC cohort, variable selection was executed using Lasso regression, CoxBoost algorithm, RSF (Random Survival Forest), StepCox (both forward and backward). Progressing from this stage, we generated 80 combinations of machine learning algorithms based on lasso regression, RSF, GBM (Gradient Boosting Machine), Survival-SVM (Support Vector Machine), SuperPC (Super Principal Component Analysis), ridge regression, plsRcox (Partial Least Squares Regression-Cox Model), CoxBoost algorithm as well as StepCox and enet methods. Ultimately, risk scores were computed using the signature derived from the training cohort in other testing cohorts such as GSE14520 and ICGC-LIHC. The optimal prognostic model was selected based on the average c-index across all cohorts.

The utilization of the prophetic package in R software expedited the calculation of half maximal inhibitory concentration (IC50) values, which are crucial for assessing the efficacy of targeted therapeutic drugs obtained from various literature sources. A wide range of algorithms, including ssGSEA, TIMER, CIBERSORT, QUANTISEQ, MCP-counter, XCELL, and EPIC were meticulously employed to evaluate the immune microenvironment and estimate the abundance of immune cells in a variety of samples. The ESTIMATE algorithm was skillfully used to determine immune scores and stromal scores as indicators of the microenvironment’s status. Additionally, a gene correlation analysis matrix was ingeniously generated using the R-ggcorrplot package with Spearman’s correlation coefficient employed for evaluating correlations.

### Specimens from patients

Patient-derived liver cancer specimens (*n* = 12) and adjacent non-tumor tissues (*n* = 12) were collected from patients undergoing treatment at the Second Affiliated Hospital of Harbin Medical University, Harbin, China. These tissues underwent reverse transcription-quantitative polymerase chain reaction (RT-qPCR) experiments for detection purposes. The research protocol was approved by the Medical Ethics Committee of the Second Affiliated Hospital of Harbin Medical University, and written informed consent was obtained from all participating patients.

### Cell culture

Human HCC cell lines and normal human liver cells were obtained from Zhongqiao Xinzhou Biotechnology Co., Ltd., Shanghai, China. The cells were cultured in Dulbecco’s modified Eagle’s medium (DMEM) supplemented with 10% fetal bovine serum (FBS) and 1% penicillin-streptomycin (Beyotime, Shanghai, China) and incubated at a temperature of 37 °C inside a 5% CO_2_ incubator.

### Transfection

The jetPRIME^®^ transfection reagent (Polyplus, America) was employed for the delivery of siRNA into the cells. The small interfering SPP1 RNA (si-SPP1) and negative control (si-NC) were obtained from RiboBio (Guangzhou, China). The human si-SPP1 gene sequences are as follows: si-SPP1-#2 (target sequence: GAACGACTCTGATGATGTA); si-SPP1-#4 (target sequence: GCCACAAGCAGTCCAGATT).

### RNA isolation and real-time quantitative reverse transcription polymerase chain reaction

Total RNA extraction was carried out using the Trizol reagent (Invitrogen, Carlsbad, CA, USA). RNA was reverse transcribed into cDNA using a reverse transcription kit (Toyobo, Japan). mRNA levels were quantified using the SYBR Green Master Mix (Toyobo, Japan), with GAPDH or 18s serving as internal controls. The primers used are listed in Supplementary Table 1.

### Western blot analysis

The extraction of proteins was carried out using Ripa buffer (Beyotime, Shanghai, China), supplemented with protease inhibitors (Roche, Switzerland) and phosphatase inhibitors (Roche, Switzerland). The protein concentration was determined utilizing the bicinchoninic acid (BCA) assay kit (Beyotime, Shanghai, China). Subsequently, proteins were separated via sodium dodecyl sulfate-polyacrylamide gel electrophoresis (SDS-PAGE), transferred onto nitrocellulose membranes, and subsequently blocked. Post-blocking, the membranes were incubated overnight at 4 °C with primary antibodies against β-actin (#bs-0061R, BIOSS, Beijing, China, 1:1000), SPP1 (#22952-1-AP, Proteintech, Wuhan, China, 1:1000), BAX (#60267-1-Ig, Proteintech, Wuhan, China, 1:1000), Bcl-2 (#A0208, ABclonal, Wuhan, China, 1:1000), Caspase-3/Cleaved Caspase-3 (#WL02117, Wanleibio, Shenyang, China, 1:1000), PKC Alpha (#21,991-I-AP, Proteintech, Wuhan, China, 1:1000), Phospho-PKC Alpha (#28926-1-AP, Proteintech, Wuhan, China, 1:1000), PI3K (#CY5355, Abways, Shanghai, China, 1:1000), Phospho-PI3K (#CY6427, Abways, Shanghai, China, 1:1000), AKT (#CY5561, Abways, Shanghai, China, 1:1000), Phospho-AKT (#CY6569, Abways, Shanghai, China, 1:1000), Phospho-mTOR (Affinity Biosciences, AF3308, USA, 1:1000), mTOR (Affinity Biosciences, AF6308, USA, 1:1000), Tubulin (#WL01931, Wanleibio, Shenyang, China, 1:1000), NDRG1 (#CY7079, Abways, Shanghai, China, 1:1000), SFN (#CY5856, Abways, Shanghai, China, 1:1000) and LDHA (#CY8276, Abways, Shanghai, China, 1:1000). After washing the membranes three times for five minutes each with TBST buffer, the membranes were incubated with anti-rabbit/mouse IgG conjugated to horseradish peroxidase (LI-COR Bioscience, Lincoln, USA) at a dilution of 1:10,000 under dark conditions for fifty minutes. Protein bands were quantified using an Odyssey infrared imaging system (LI-COR, Lincoln, NE, USA). β-Actin served as an internal control reference.

### EdU

The Huh7/HCCLM3 cells were incubated with EdU in a culture medium containing 300 µl of EdU reagent, at a concentration of 50 µmol/L, for 2 h. Post-incubation, the cells underwent washing, fixation, and decolorization, followed by permeabilization with 300 µl of 0.5% Triton X-100. Subsequently, the cells were stained with Apollo for DNA staining and visualized using a confocal laser scanning microscope (FV300, Olympus, Japan).

### TUNEL

For TUNEL transfection, Huh7/HCCLM3 cells were cultured in ultra-low attachment plates (Corning) at a density of 1 × 10^5^ cells per well. The evaluation of cell apoptosis was conducted employing the terminal deoxynucleotidyl transferase dUTP nick-end labeling (TUNEL) method. Cells were fixed with 4% paraformaldehyde and permeabilized with 0.1% Triton X-100. The Huh7/HCCLM3 cell mixture and TUNEL reaction solution were incubated at 37 °C in darkness for one hour, followed by DAPI staining for 15 min. Fluorescence detection of the cells was performed using a confocal laser scanning microscope (FV300, Olympus, Japan).

### Cell viability, caspase 3 activity assay, and annexinV/propidium idide staining

After transfection, Huh7/HCCLM3 cells were then cultured in ultra-low attachment plates (Corning) at a density of 1 × 10^6^ cells/well. At designated time points, suspended cells were collected and subjected to cell viability analysis using the Trypan Blue Staining Cell Viability Assay Kit (Beyotime, Shanghai, China), Caspase 3 Activity Assay Kit (Beyotime, Shanghai, China), and AnnexinV-FITC/PI Apoptosis Detection Kit (Meilunbio, Dalian, China), following their respective experimental protocols. Trypan Blue staining was conducted using a hemocytometer and cell counter, while AnnexinV-FITC/PI was employed for flow cytometry analysis.

### ELISA

The supernatant of the cell culture medium was collected and subjected to centrifugation at 1000 g for 20 min at 4 °C to remove impurities and cellular debris. Subsequently, the levels of SPP1 were quantified using a commercially available ELISA kit (Elabscience, Wuhan, China), following the manufacturer’s instructions.

### Wound healing assay

The cells that had received transfection were cultured with serum-free medium in a 6-well plate. Subsequently, the alterations in wound area over the course of 0 to 24 h subsequent to injury induction using the damaged tip of a 10 µl pipette were documented at ×100 magnification under a microscope (Olympus, Japan).

### Transwell assay

In the transwell migration experiment, cells were suspended in serum-free culture medium. A 200 µL cell suspension was then transferred to the upper chamber (Corning, America), which contained an 8 μm polycarbonate filter (BD, America) (5 μm filter for MDSCs and Tregs). The lower chamber was filled with 500 µL of culture medium supplemented with 10% fetal bovine serum (the serum-free culture medium supplemented with recombinant SPP1 protein for MDSCs and Tregs). After a 24-hour incubation, the cells that had invaded the lower chamber were fixed using cold methanol and stained with crystal violet. The migration of cells was observed under a microscope (Olympus, Japan) at ×100 magnification, whereas MDSCs and Tregs were quantified using a hemocytometer and cell counter.

### Animal experiments

We generated lentiviral vectors containing shSPP1 (target sequence: CGAGGAGTTGAATGGTGCATA) and a control vector (shNC), which were packaged with slow virus. Stable expression of shSPP1 or shNC was achieved in 2 × 10^6^ Huh7 cells suspended in a high-concentration matrix of 200 µL. Male BALB/c nude mice aged 4–5 weeks without thymus were randomly divided into three groups (6–10 mice/group). Each mouse received a tail vein injection of 200 µL cell suspension, and their body weight changes were monitored. In the drug treatment group, oxaliplatin (5 mg/kg) was administered via the tail vein every three days after injecting tumor cells for two weeks. After seven weeks, D-Luciferin (Solarbio, Beijing, China) was intraperitoneally injected to monitor metastasis using the IVIS@ Lumina II system (Caliper Life Sciences, Hopkinton, MA, USA). The lungs of euthanized mice from each group were isolated and subjected to H&E staining and immunofluorescence staining after fixation.

### Isolation of primary murine cells (MDSCs/Tregs)

MDSCs were isolated using the recommended medium (#20,144, STEMCELL Canada), which was used for flushing bone marrow cells from mouse femurs and tibias. Tregs were isolated from mouse spleens using the same medium, followed by filtration through a 70 μm nylon mesh to remove impurities and tissue debris. MDSCs were extracted using the Mouse MDSC (CD11b + Gr1+) Isolation Kit (#19,867, STEMCELL Canada), while Tregs were extracted using the Mouse CD25 Regulatory T Cell Positive Selection Kit (#18,782, STEMCELL Canada).

### Flow cytometry

The treated cells were collected, suspended in a centrifuge tube, and then transferred into a flow tube containing the cell suspension (1 × 10^6^-1 × 10^7^). MDSCs were labeled with CD11b (#FHF011b, 4 A Biotech, Beijing, China) and Gr1 (#FMALY6GC, 4 A Biotech, Beijing, China), while Tregs were labeled with CD4 (#100,509, Biolegend, USA) and FOXP3 (#126,409, Biolegend, USA), following pre-addition to disrupt the membrane. Flow cytometry was employed for detection.

### Statistical analysis

All data were presented as the mean ± SD of at least three independent experiments. Student’s unpaired two-tailed *t* test was employed for two-group comparisons, while one-way analysis of variance (ANOVA) followed by Dunnett’s corrected post hoc correction was utilized for multigroup comparisons. The analyses were conducted using GraphPad Prism 9.0 software (GraphPad Software, San Diego, CA, USA). Statistical significance was considered for *p* value < 0.05.

## Results

### Elevated expressions of ARGs are substantially correlated with a deteriorated prognosis in HCC

**Fig. 1:**
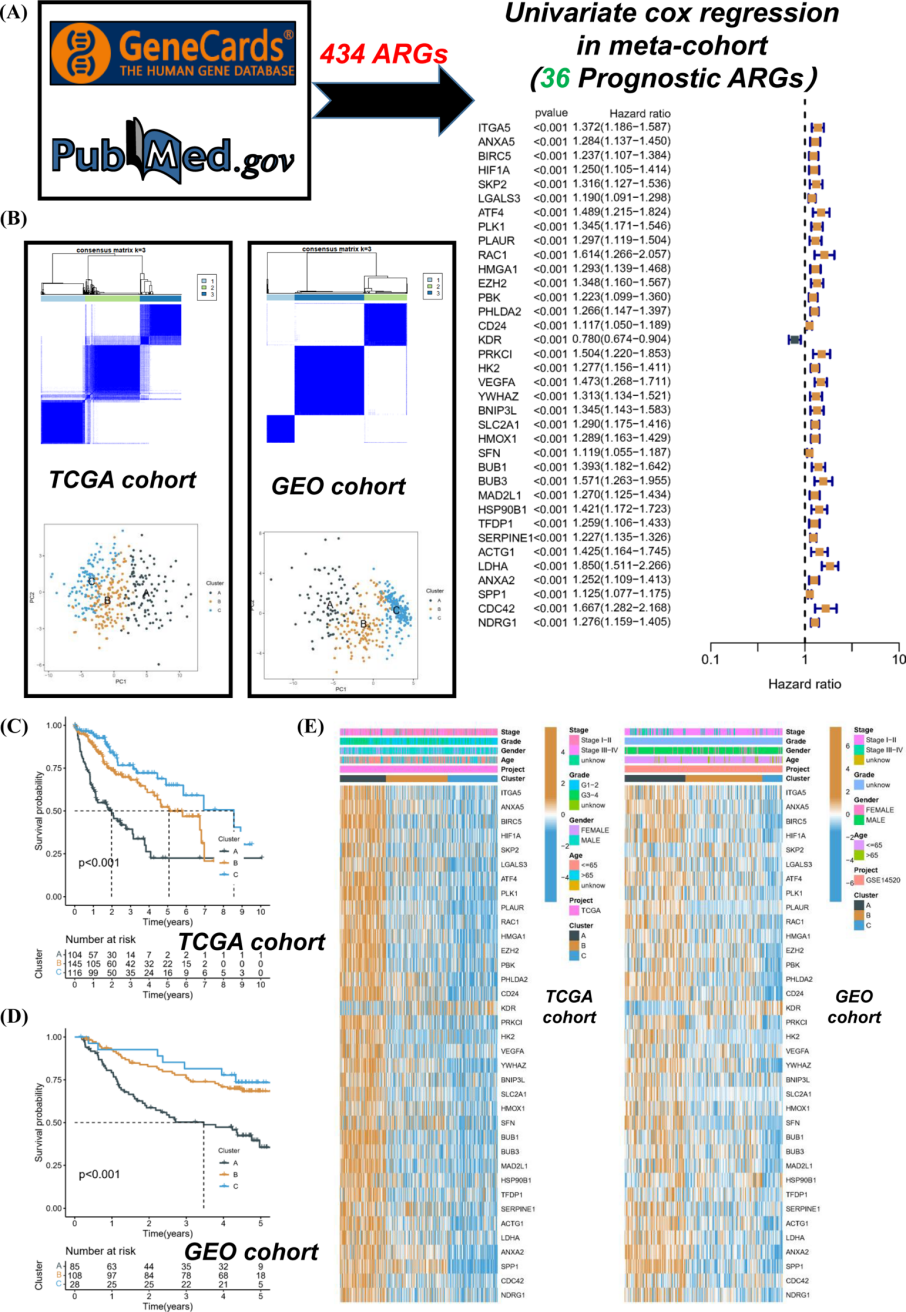
Elevated expressions of ARGs are substantially correlated with a deteriorated prognosis in HCC. (**A**) The ARGs related to prognosis were screened in HCC. (**B**) Unsupervised classification of TCGA-LIHC and GSE14520 cohorts was performed according to the expressions of ARGs. (**C** and **D**) The survival differences of different subtypes were compared. (**E**) The clinical characteristics and prognostic ARGs distribution of different subtypes were compared

The initiation of our study involved conducting univariate Cox regression analysis on ARGs, which led to the identification of 36 ARGs that exhibited significant prognostic value (Fig. 1A). Subsequently, based on the expression levels of ARGs, we performed unsupervised classification on patients from the TCGA-LIHC and GSE14520 cohorts. The optimal classification was achieved when the K value equaled 3, leading to the identification of two distinct molecular subtypes (Fig. 1B). PCA analysis revealed relative discreteness among the three molecular subtypes. Survival analysis in the TCGA cohort indicated that subtype A exhibited a poorer prognosis, while subtype C demonstrated the best survival outcome (Fig. 1C). This observation was consistent in the GEO cohort as well (Fig. 1D). Heatmaps depicted clinical characteristics and distribution patterns of prognostic ARGs across different clusters. Intriguingly, most ARGs were significantly upregulated in subtype A but downregulated in subtype C (Fig. 1E). In essence, the present findings robustly confirmed a significant correlation between the expression of ARGs and HCC prognosis, consistently emphasizing that elevated levels of ARGs are frequently associated with unfavorable prognostic outcomes.

### Multiple signaling pathways are involved in anoikis resistance in HCC

**Fig. 2:**
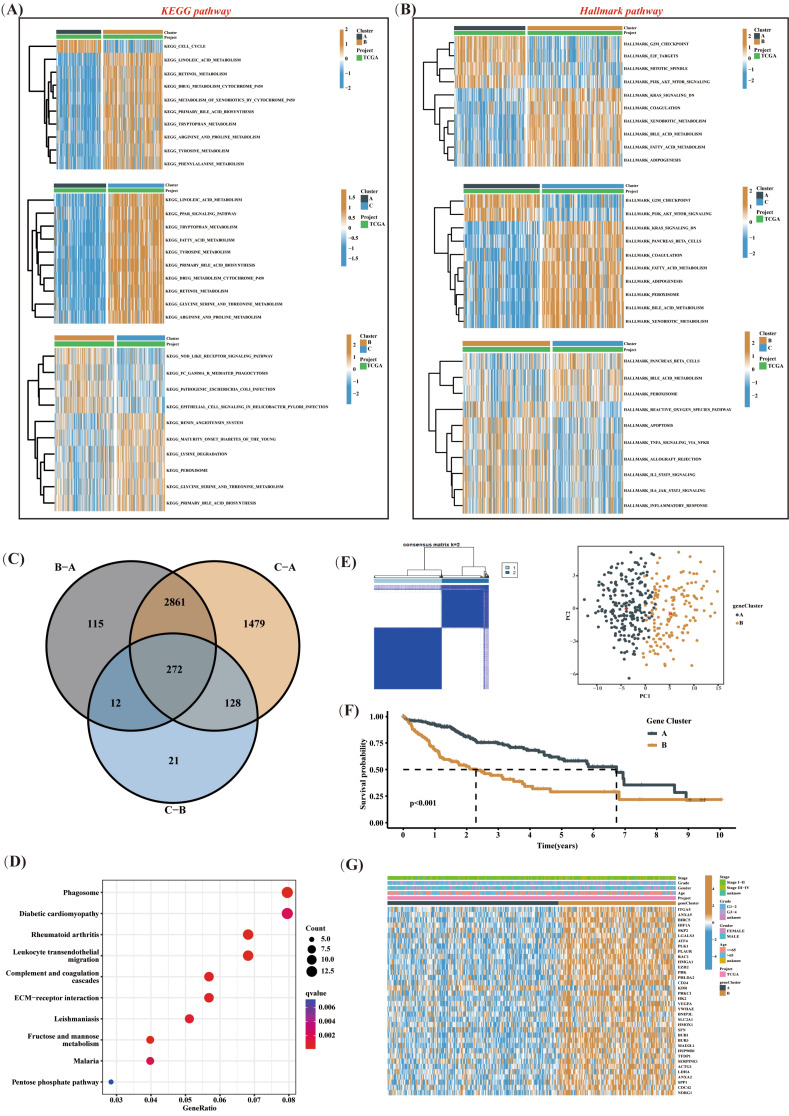
Multiple signaling pathways are involved in anoikis resistance in HCC. (**A** and **B**) GSVA enrichment analysis was performed to compare HALLMARK/KEGG pathways between different subtypes. (**C**) Common DEGs among different subtypes were identified. (**D**) Enrichment analysis of DEGs was performed. (**E**) Unsupervised classification of the TCGA-LIHC cohort was performed based on the expressions of DEGs associated with prognosis. (**F**) The survival differences of different subtypes were compared. (**G**) The clinical characteristics and prognostic ARGs distribution of different subtypes were compared

To delve into the factors shaping diverse prognoses, we conducted an in-depth analysis of mutation spectra across various subtypes. Our research uncovered a notably lower proportion of TP53 mutations in subtype C, in comparison to subtypes B and A within the top 10 genes. Conversely, subtype A exhibited a significantly reduced frequency of CTNNB1 mutations compared to subtypes C and B (Supplementary Figure S1A). Subsequently, we carried out GSVA enrichment analysis and detected significant deviations in HALLMARK/KEGG pathways associated with various cancer occurrences among different subtypes (Fig. 2A-B). Particularly, both subtype B and C demonstrated a notable suppression of the PI3K/AKT/mTOR signaling pathway in comparison to subtype A. We further conducted a comparative analysis between the two subtypes and identified 272 genes (Fig. 2C and Supplementary Figure S1B-D) that exhibited differential expression in both subtypes. The pathways associated with these genes were primarily involved in biological processes, such as phagosome and ECM-receptor interaction (Fig. 2D). By implementing single-factor Cox regression analysis, we determined prognostic genes based on the aforementioned differentially expressed genes (DEGs). When classifying patients according to the expression of these prognostic genes, an optimal classification was achieved with a K value of 2, ultimately revealing two distinct regulatory subtypes (Fig. 2E). Among these subtypes, subtype A exhibited the most favorable prognosis (Fig. 2F). Intriguingly, heatmap analysis disclosed varying expression levels of ARGs among different subtypes, with subtype B exhibiting higher levels of expression (Fig. 2G). These findings provided evidence supporting divergent prognoses for various subtypes and suggested the involvement of various signaling pathways, such as the PI3K/AKT/mTOR axis, in resistance to anoikis of HCC. Furthermore, our results corroborated the association between high expression of ARGs and poor prognosis.

### Immunity is closely related to anoikis in HCC

**Fig. 3:**
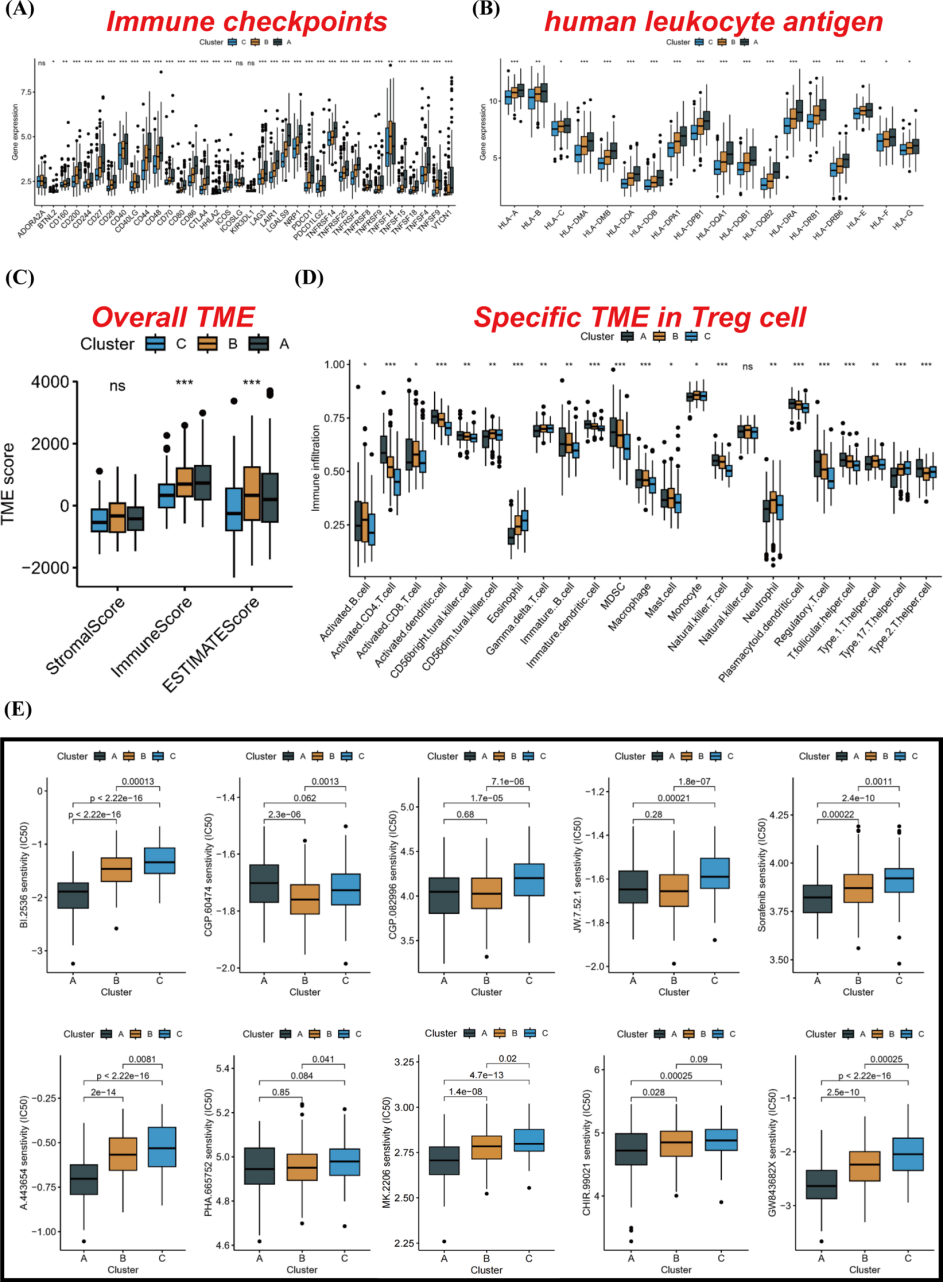
Immunity is closely related to anoikis in HCC. (**A** and **B**) Based on the TCGA-LIHC cohort, the expressions of HLA and ICI mRNA from different subtypes were compared. (**C**) The ESTIMATE algorithm was used to compare TME score among different subtypes. (**D**) The infiltration status of various immune cells in different subtypes was compared by ssGSEA analysis. (**E**) The IC50 values of common targeted drugs in different subtypes were compared. *p < 0.05, **p < 0.01, and ***p < 0.001

Subsequently, we conducted a comparative analysis of human leukocyte antigen (HLA) and immune checkpoint inhibitor (ICI) mRNA expression levels across distinct molecular subtypes. Intriguingly, subtype A exhibited elevated mRNA expression in the majority of HLA and ICI genes (Fig. 3A-B). The ESTIMATE algorithm also corroborated that subtype A had a higher immune score compared to subtype C (Fig. 3C). Moreover, our ssGSEA analysis enabled a more detailed characterization of the composition of various immune cells in different samples. Box plots depicted the tumor microenvironment (TME) status among different molecular subtypes, highlighting a significant upregulation of Tregs and MDSCs in subtype A. This finding might provide an explanation for the inferior prognosis observed in some ‘hot tumor’ states associated with subtype A (Fig. 3D). Additionally, we predicted IC50 values for various commonly used targeted drugs and discovered that subtype A was more sensitive in most cases (Fig. 3E). In summary, these findings revealed a robust correlation between immunity and ARGs, wherein Tregs and MDSCs assumed substantial roles within the immune microenvironment.

### SFN, LDHA, SPP1 and NDRG1 are the core anoikis related prognostic genes in HCC

**Fig. 4:**
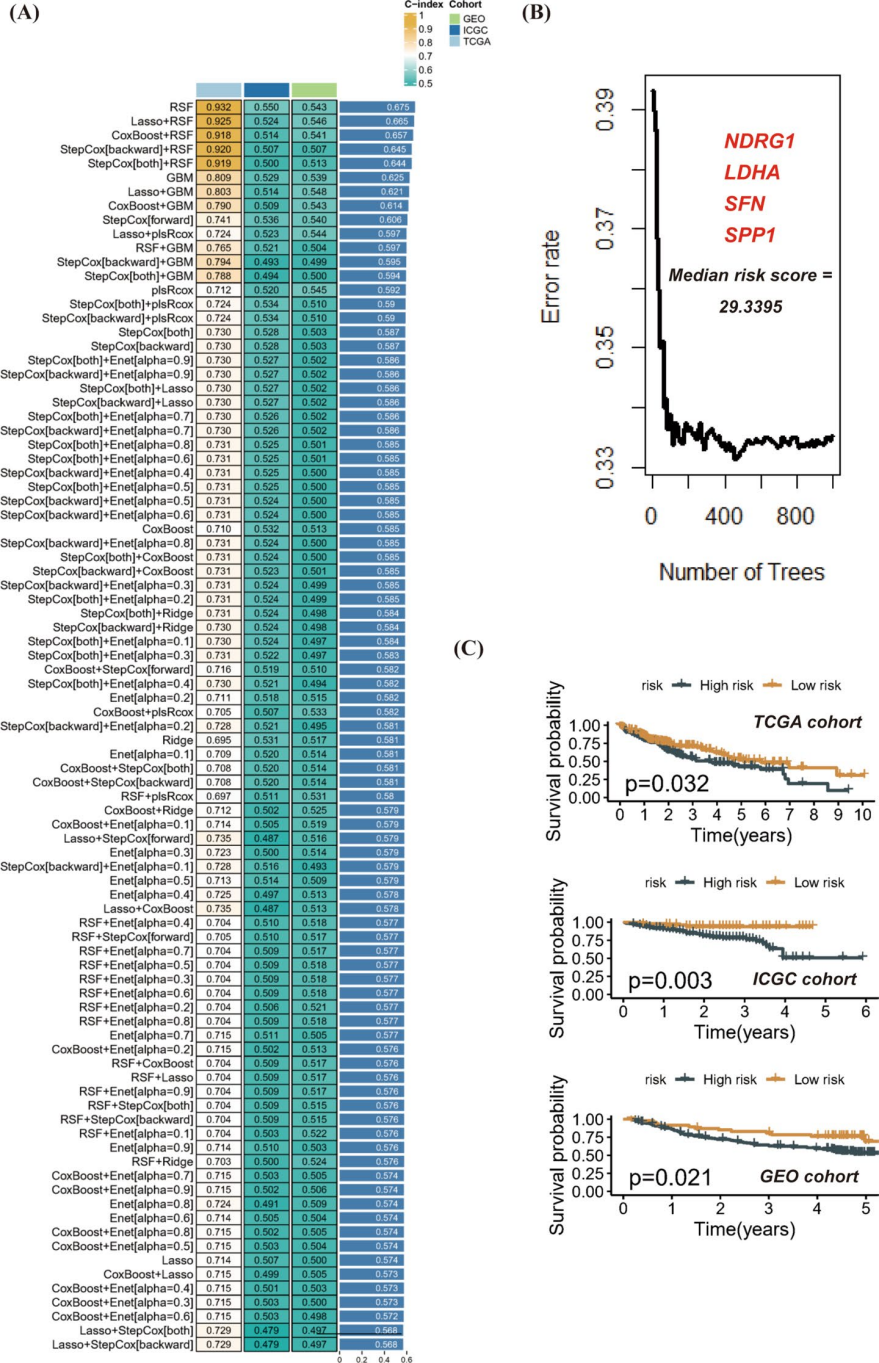
SFN, LDHA, SPP1 and NDRG1 are the core anoikis related prognostic genes in HCC. (**A**-**B**) Eighty combinations of machine learning algorithms were created to select the best prognostic model based on the average C-index of each cohort. (**C**) According to the risk prognosis model, the survival differences between high and low risk groups in multiple cohorts were compared

The development of a prognostic model for patients diagnosed with HCC was preceded by the strict adherence to the established pipeline. Employing the TCGA-LIHC cohort for the machine learning algorithm’s modeling, and incorporating the GSE14520 and ICGC-LIHC cohorts for validation, led to the identification of RSF as the optimal prognostic model. This decision was based on the average C-index across all cohorts, and four prognosis-related ARGs were incorporated into the modeling process (Fig. 4A-B). Furthermore, this model’s validity was reinforced by its successful validation in multiple platform cohorts. A cut-off value of 44.38 from the TCGA cohort was chosen to effectively stratify all patients into high-risk and low-risk groups. This model’s effectiveness in discriminating patient survival outcomes was notably demonstrated in the TCGA, ICGC, and GEO cohorts (Fig. 4C).

To investigate the expression patterns of SFN, LDHA, SPP1, and NDRG1 genes in various samples within the RSF model, we created a heatmap. This heatmap revealed that all four genes exhibited heightened expression levels in the high-risk group of the validation set (Supplementary Figure S2A). Additionally, the receiver operating characteristic (ROC) curves confirmed that the risk score demonstrated a robust predictive ability for survival in both the GEO and ICGC cohorts (Supplementary Figure S2B). To explore the differences in clinical characteristics between the high- and low-risk groups, we conducted a comparative analysis of staging and grading in different risk groups within the TCGA cohort. The results uncovered significant disparities in these clinical features across various risk groups (Supplementary Figure S2C). Moreover, we performed univariate and multivariate Cox regression analyses on the risk score using both the GEO and ICGC cohorts. The findings confirmed that the risk score served as an independent prognostic factor (Supplementary Figure S2D). The findings implied that SFN, LDHA, SPP1, and NDRG1 served as the fundamental target genes.

### The SPP1 protein enhances anoikis resistance and malignant behaviors of HCC cells

Based on the comprehensive exome sequencing data, no statistically significant disparities were detected in the top mutated genes, MUC16 and TTN, between high-risk and low-risk patients (Supplementary Figure S3A). Additionally, we scrutinized the expression patterns of SFN, LDHA, SPP1, and NDRG1 genes across various samples. Tumor samples consistently exhibited elevated expression levels of SFN, SPP1, and NDRG1 genes, regardless of being paired or unpaired, in comparison to non-tumor samples. However, LDHA did not exhibit any significant variations in expression across different sample types (Supplementary Figure S3B-C). In the TCGA-LIHC cohort analysis, only one sample displayed a mutation in the NDRG1 gene (Supplementary Figure S3D). Supplementary Figure S3E illustrates potential drug targets associated with these four genes. Furthermore, NDRG1 primarily exhibited copy number amplification, whereas SFN predominantly showed copy number deletion (Supplementary Figure S3F). Protein-protein interaction(PPI) network analysis unveiled interactions among these four genes (Supplementary Figure S3G).

**Fig. 5:**
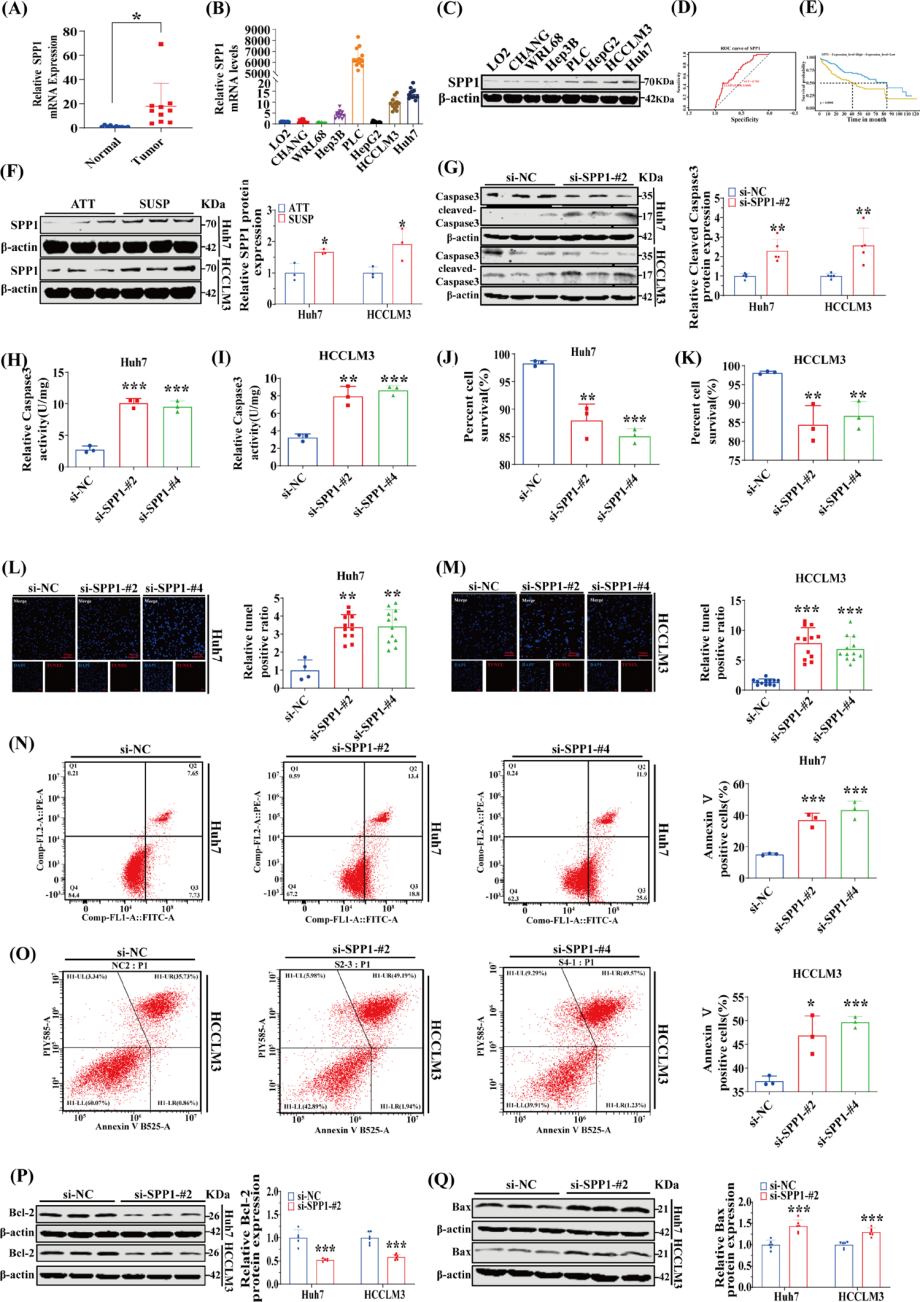
The SPP1 protein enhances anoikis resistance and malignant behaviors of HCC cells. (**A**) The mRNA levels of SPP1 in HCC and non-cancerous tissues were compared (*n* = 10). (**B**) The expression levels of SPP1 mRNA in different HCC cell lines and normal liver cell lines were compared (*n* = 11–12). (**C**) The expression levels of SPP1 protein in different HCC cell lines and normal liver cell lines were compared. (**D** and **E**) Based on the TCGA-LIHC cohort, the prognostic survival analysis of SPP1 was performed. (**F**) Hepatoma Huh7 and HCCLM3 cells were cultured under attached (ATT) or suspension (SUSP) conditions for 48 h, and SPP1 expression levels were determined by Western blot analysis (*n* = 3). (**G**) SPP1-knockdown Huh7 and HCCLM3 cells were cultured in suspension for 48 h, and the expression of cleaved caspase-3 was compared with that of control cells by Western blotting (*n* = 5). (H and I) SPP1-knockdown Huh7 and HCCLM3 cells were cultured in suspension conditions for 48 h. The anoikis activity was compared by caspase-3 activity assay (*n* = 3). (J and K) After SPP1-knockdown Huh7 and HCCLM3 cells were cultured in suspension for 48 h, cell viability was compared by trypan blue staining (*n* = 3). (**L** and **M**) After SPP1-knockdown Huh7 and HCCLM3 cells were cultured in suspension for 48 h, apoptosis of cells was compared by TUNEL (*n* = 3). (**N** and **O**) After SPP1-knockdown Huh7 and HCCLM3 cells were cultured in suspension for 48 h, apoptosis of cells was compared by flow cytometry using Annexin V/PI staining kit (*n* = 3). (**P** and **Q**) After SPP1-knockdown Huh7 and HCCLM3 cells were cultured in suspension for 48 h, the expressions of Bcl-2 and Bax were compared by Western blot (*n* = 5–6). *p < 0.05, **p < 0.01, and ***p < 0.001

To further delineate the crucial target gene, we initially conducted a comparative analysis of SPP1, NDRG1, SFN and LDHA mRNA expression levels in clinical samples of HCC and adjacent non-tumor tissues, as well as various HCC cell lines and normal liver cell lines. Our findings revealed a significant upregulation of SPP1 in both HCC tissues and HCC cell lines (Fig. 5A-B; Supplementary Figures S4A-B). Additionally, we compared the protein expression levels of these four core genes between human HCC tissues and adjacent non-tumorous tissues. The results revealed that SPP1 expression was significantly upregulated in HCC tissues, while NDRG1, SFN, and LDHA were also upregulated in the majority of HCC tissues (Supplementary Figures S4C-F). Subsequently, the protein expression level of SPP1 was validated through western blot analysis in an array of human HCC cell lines (Fig. 5C). Guided by the differential expression pattern observed for SPP1, we opted for the Huh7 and HCCLM3 cells for targeted silencing experiments. Moreover, ROC curve analysis and KM survival analysis utilizing data from the TCGA-LIHC dataset corroborated that SPP1 holds promise as a prognostic diagnostic value (Fig. 5D-E). Next up, we initially examined the expression of SPP1 protein between attached and suspended cells. Compared to adherent cells, the level of SPP1 was notably increased in suspended cells (Fig. 5F), suggesting a potential role for SPP1 in conferring resistance against anoikis in HCC. To further explore its anti-anoikis function, we effectively suppressed SPP1 expression as demonstrated in Supplementary Figure S5A-D. Subsequently, we investigated the impact of altered SPP1 expression on anoikis resistance. Similar to other forms of programmed cell death, caspase-3 cleavage activation often characterizes anoikis [5]. To further delve into the impact of SPP1 on programmed cell death in suspended cells, we conducted a western blot analysis to ascertain the degree of caspase-3 cleavage. Our research revealed a notable escalation in the level of cleaved caspase-3 in suspended cells subsequent to the knockdown of SPP1 (Fig. 5G). We discovered that the inhibition of SPP1 expression significantly elevated the rate of anoikis after suspending cell culture for 48 h. This was evidenced by caspase-3 activity assay (Fig. 5H-I), trypan blue staining assay for cell viability detection (Fig. 5J-K), TUNEL apoptosis detection assay (Fig. 5L-M), and Annexin V/PI staining assay (Fig. 5N-O). Furthermore, the repression of SPP1 resulted in a significant downregulation of the anti-apoptotic protein Bcl2, and correspondingly, a substantial upregulation of the pro-apoptotic protein Bax during suspension growth for Huh7 and HCCLM3 cells (Fig. 5P-Q and Supplementary Figure S6A-B). The existing literature indicated that cancer cells, which exhibit resistance to anoikis, also display exacerbated malignant characteristics that accelerate cancer progression in the absence of anchorage sites [13, 14]. Subsequently, we examined the influence of SPP1 expression on cellular proliferation and migration. Employing the EdU cell proliferation assay, scratch assay, and transwell migration assay, we observed a notable suppression of both Huh7 and HCCLM3 cells’ proliferation and migration following SPP1 knockdown (Supplementary Figure S7A-F). Collectively, these findings suggested that SPP1 augmented anoikis resistance and nurtured malignant traits in HCC cells.

### The SPP1 protein activates the PI3K/AKT/mTOR signaling pathway through PKCα phosphorylation to resist anoikis in HCC

**Fig. 6:**
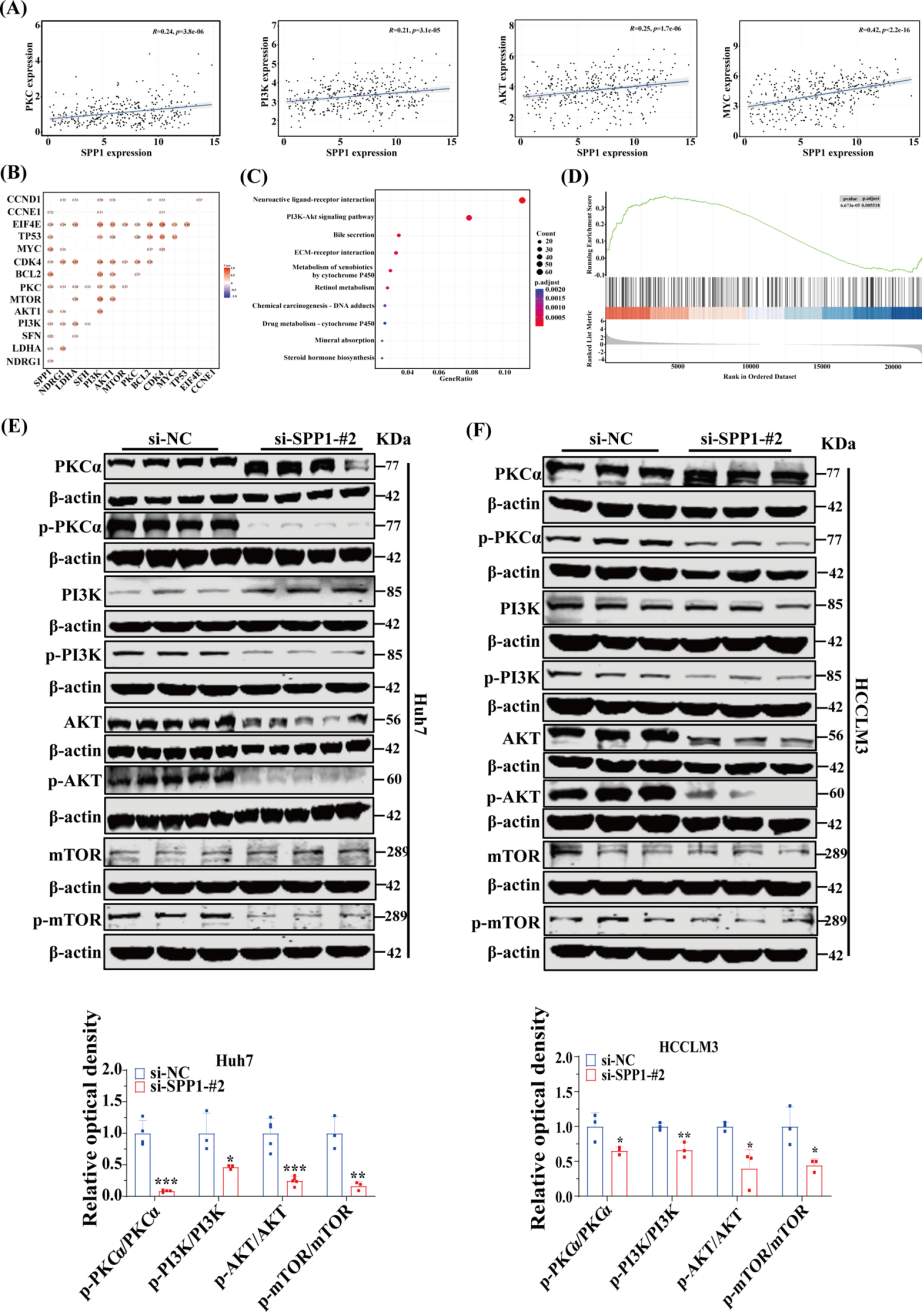
The SPP1 protein activates the PI3K/AKT/mTOR signaling pathway through PKCα phosphorylation to resist anoikis in HCC. (**A** and **B**) The correlation between SPP1 and PKC, as well as the PI3K/AKT/mTOR signaling pathway, was analyzed using the Spearman correlation coefficient. (**C** and **D**) Based on the TCGA-LIHC cohort, KEGG and GSEA enrichment analyses were performed according to the expression of SPP1. (**E** and **F**) After SPP1-knockdown Huh7 and HCCLM3 cells were cultured in suspension for 48 h, the expressions of phospho-PKC, phospho-PI3K, phospho-AKT and phospho-mTOR were compared by Western blot (*n* = 3–5). *p < 0.05, **p < 0.01, and ***p < 0.001

We further investigated the mechanism by which SPP1 mediated anoikis resistance and metastasis in HCC. Studies have reported that SPP1 can activate the PI3K/AKT/mTOR signaling pathway through PKCα phosphorylation, a pathway closely associated with tumor invasion and metastasis [15, 16]. The robust association between SPP1 and the PKC, as well as the PI3K/AKT/mTOR signaling pathways, was unequivocally revealed by gene correlation analysis. SPP1 was further identified as a pivotal player among four target genes (Fig. 6A-B). KEGG and GSEA enrichment analyses of SPP1 corroborated the significant enrichment in the PI3K/AKT/mTOR signaling pathway, and its positive correlation with this pathway was also demonstrated (Fig. 6C-D). The knockdown of SPP1 notably reduced the phosphorylation levels of PKCα and PI3K/AKT/mTOR in suspended cells (Fig. 6E-F, Supplementary Figure S8A-B). This suggested that SPP1, through mediating integrin-induced PKCα phosphorylation, activated the PI3K/AKT/mTOR signaling pathway, thereby conferring resistance against anoikis.

### The SPP1 protein recruits MDSCs and Tregs and affects their functions

**Fig. 7:**
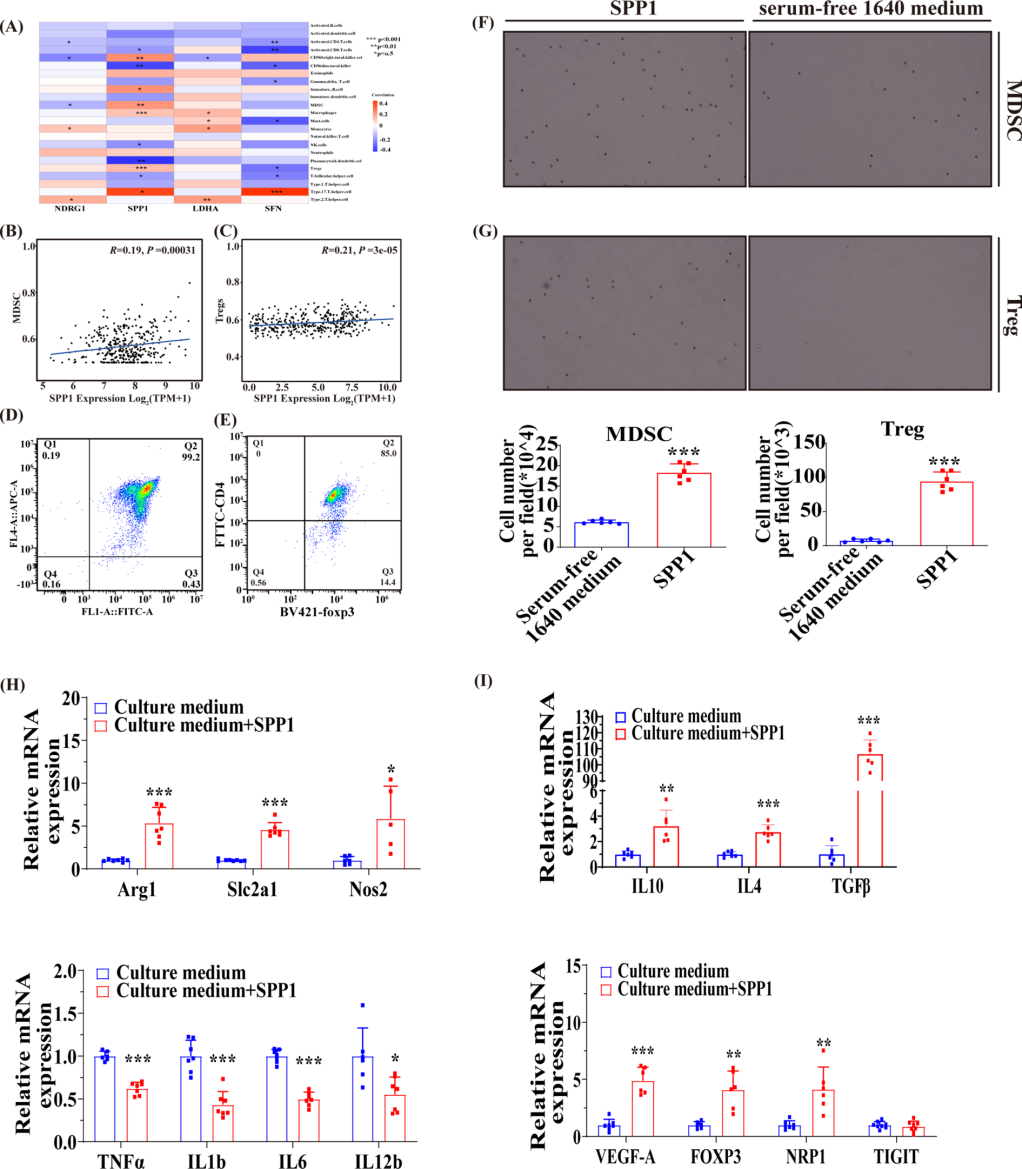
The SPP1 protein recruits MDSCs and Tregs and affects their functions. (**A**, **B** and **C**) The associations between SFN, LDHA, SPP1, and NDRG1 with immune cells were investigated. (**D** and **E**) The extracted MDSCs and Tregs were identified by flow cytometry. (**F** and **G**) The migratory behaviors of MDSCs and Tregs were assessed following stimulation with mouse recombinant SPP1 protein (*n* = 6). (**H** and **I**) The expressions of characteristic genes in MDSCs and Tregs were assessed following stimulation with mouse recombinant SPP1 protein (*n* = 5–8). *p < 0.05, **p < 0.01, and ***p < 0.001

The profound analysis revealed a robust correlation between immunity and ARGs. Similarly, immunity plays a crucial role in tumor metastasis [9]. Initially, we examined the correlation between four target genes and various immune cells. Remarkably, SPP1 exhibited a robust positive correlation with MDSCs and Tregs (Fig. 7A-C). We further scrutinized whether SPP1 functioned as a key regulator of MDSCs and Tregs migration. Previous studies have indicated that prostate tumor cells secrete SPP1 protein to attract infiltrating MDSCs [17]. We isolated MDSCs and Tregs from the bone marrow and spleen of C57 mice using sorting reagents, followed by flow cytometry identification (Fig. 7D-E). Subsequently, transwell migration assays confirmed that recombinant mouse SPP1 protein could facilitate the migration of both MDSCs and Tregs (Fig. 7F-G). Moreover, stimulation with mouse-derived recombinant SPP1 protein induced the expression of pro-tumor genes (such as Arg1, Slc2a1, and Nos2) in MDSCs while repressing the expression of anti-tumor genes (such as Il1b, Tnfa, and Il12b) (Fig. 7H). Similarly, characteristic genes expression were activated in Tregs (Fig. 7I). These findings suggested that HCC cell-secreted SPP1 protein can attract infiltrating MDSCs and Tregs while boosting their immunosuppressive effects.

### Downregulation of SPP1 suppresses HCC metastasis and MDSCs infiltration in vivo

**Fig. 8:**
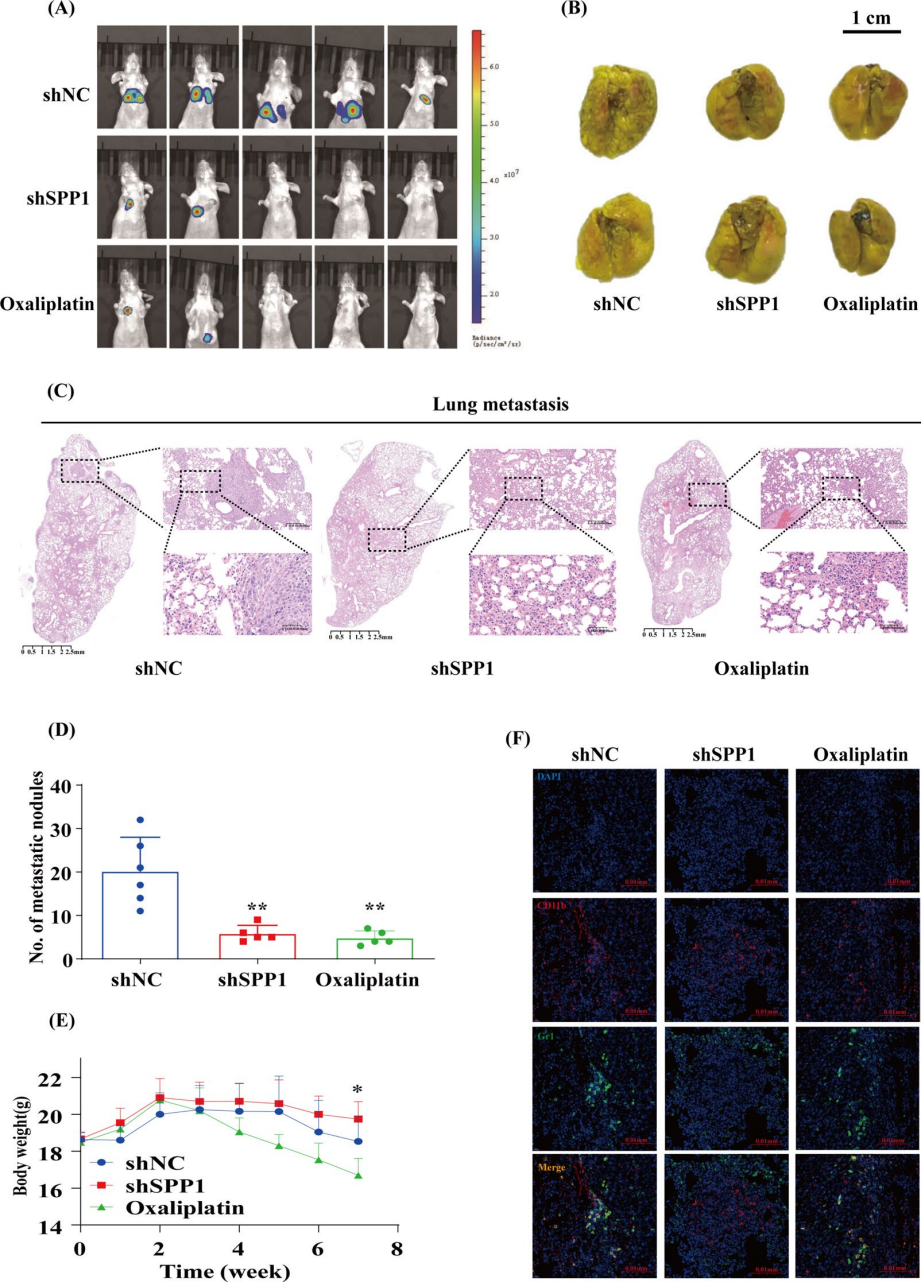
Downregulation of SPP1 suppresses HCC metastasis and MDSCs infiltration in vivo. (**A**) Nude mice received intravenous injections of shNC or shSPP1 Huh7 cells, followed by the capture of bioluminescent images after a period of 7 weeks. The shNC group consisted of both the shNC and oxaliplatin groups (*n* = 5). (**B**) Representative images of lung metastases were shown (*n* = 5–6). (**C**) Representative H&E stained images of lungs were obtained from the shNC, shSPP1, and oxaliplatin groups. The scale bars in H&E stained tissue images were 2500 μm, 200 μm, and 50 μm, respectively (*n* = 5–6). (**D**) The number of pulmonary metastatic nodules in each group was compared according to the results of H&E staining (*n* = 5–6). (**E**) The variation in body weight among each group was assessed subsequent to the injection of cells through the tail vein (*n* = 5–7). (**F**) Immunofluorescent staining for CD11b and Gr1 was conducted on lung samples from three groups: shNC, shSPP1, and oxaliplatin. The scale bars in the stained tissue images measured 20 μm (*n* = 4). *p < 0.05 and **p < 0.01

To further elucidate the pivotal role of SPP1 in anti-apoptosis in vivo, we conducted a metastasis experiment employing Huh7 cells expressing either shNC or shSPP1, supplemented with a luciferase reporter. The validation of knockdown efficiency was demonstrated in Supplementary Figure S9A. As an ecreted protein [18, 19], SPP1 was assessed in the culture medium supernatant through an ELISA kit and observed a reduction following knockdown (Supplementary FigureS9B). The control and knockdown cells were intravenously injected into BALB/c nude mice to assess the involvement of SPP1 in distant metastasis of HCC cells. Upon completion of 7 weeks, mice injected with shNC Huh7 cells exhibited a notably heightened luciferase activity in comparison to those injected with shSPP1 cells, while no significant disparity was observed between the shSPP1 group and the oxaliplatin group (Fig. 8A). Subsequently, the mice were euthanized to facilitate the analysis of lung metastatic nodules. Mice injected with shNC cells manifested a significantly higher count of lung metastatic nodules in comparison to the other two groups (Fig. 8B, Supplementary Figure S9C). In consonance, H&E staining results corroborated these findings (Fig. 8C, Supplementary Figure S9D). After conducting a comparison of the disparities in the number of metastatic nodules among diverse groups of nude mice based on H&E staining outcomes, we observed a notably significant elevation in the incidence of metastatic nodules within the shNC group in comparison to the other two groups (Fig. 8D, Supplementary Figure S9D). Furthermore, a considerable decrease in body weight was detected in the oxaliplatin group without in the shSPP1 group (Fig. 8E). Upon conducting immunofluorescence analysis, we discovered a substantial reduction in MDSCs infiltration within the shSPP1 group (Fig. 8F). Collectively, these findings implied that SPP1 played a vital role in promoting HCC metastasis and mediating immune evasion in vivo.

The schematic model depicts the mechanism by which SPP1 influences resistance to anoikis and promotes metastasis in HCC. The secretion of SPP1 by HCC cells activates the PI3K/AKT/mTOR signaling pathway through integrin-mediated PKCα phosphorylation, thereby enhancing resistance to anoikis. Additionally, the secreted SPP1 attracts MDSCs and Tregs, facilitating their immunosuppressive function and promoting immune evasion.

## Discussion

The pivotal role of anti-anoikis in tumor metastasis has been extensively explored in various primary tumors and tumor cell lines [20, 21]. Similarly, investigations into anti-anoikis are not infrequent in HCC research [5, 22]. However, the specific mechanism underlying this phenomenon remains obscure. By integrating bioinformatics analysis with fundamental experiments, we have identified SPP1 as a crucial gene specifically involved in anoikis resistance within HCC. The relationship between SPP1 and HCC can be summarized as follows, the interaction between SPP1 + macrophages and cancer-associated fibroblasts within the HCC microenvironment established a tumor immune barrier that impeded the efficacy of immunotherapy [23]. Secreted by cancer-associated fibroblasts, SPP1 enhanced resistance to sorafenib and lenvatinib in HCC [15]. As a DNA methylation-driven gene, SPP1 accelerated the progression of HCC [24]. In summary, SPP1 plays a pivotal role in fostering HCC progression. Nevertheless, the underlying mechanism of SPP1 in HCC anoikis remains unexplored via basic experimental research. After conducting relevant bioinformatics analyses, we continued to focus on the function of SPP1 in HCC and identified that SPP1 mediated anoikis resistance through the PI3K/AKT/mTOR signaling pathway. In summary, our experimental research is the first to uncover the mechanism of SPP1 in HCC anoikis resistance, and it is the inaugural study to reveal the role of SPP1 in mediating immune escape in HCC by recruiting MDSCs and Tregs.

The prodigious resistance of cancer cells against anoikis is propelled by complex molecular mechanisms, which include the upregulation of integrin signaling, the activation of pro-survival signals, and the overexpression of growth factor receptors [25]. The PI3K/AKT/mTOR signaling axis meticulously orchestrates a multitude of cancer traits such as cell cycle progression, survival, differentiation, proliferation, migration, metabolism, and genetic stability [26]. Not only does this signaling axis play a pivotal role in growth factor-mediated actions [27], but it also exhibits a striking correlation with integrin expression levels [28, 29]. In the realm of ovarian cancer research, the involvement of the PI3K/AKT/mTOR signaling axis in anti-anoikis resistance has been unraveled [30], yet its role in liver cancer remains unexplored. Through bioinformatics analysis probing potential mechanisms underlying disparate prognoses in HCC patients, we unearthed a robust association between ARGs-induced differential prognosis and the activation of the PI3K/AKT/mTOR signaling axis. Elevated expression levels of ARGs were reciprocally associated with the activation of the PI3K/AKT/mTOR signaling axis and prognosticated a poorer outcome in HCC. Similarly, we observed a significant correlation between SPP1 and the PI3K/AKT/mTOR signaling axis, the former of which was positively correlated with downstream molecules such as MYC [31, 32]. These findings robustly corroborate our conclusion that SPP1 is a key target gene implicated in anti-anoikis resistance in HCC. Importantly, our mechanistic studies unequivocally demonstrated that the knockout of SPP1 effectively repressed PKCα phosphorylation, thereby curtailing the phosphorylation levels of PI3K, AKT, and mTOR. Prior research indicated that the SPP1 protein secreted by cancer-associated fibroblasts augmented drug resistance by activating the PI3K/AKT/mTOR signaling pathway through integrin-mediated PKCα phosphorylation [15]. This further corroborates our experimental findings. Moreover, the extracellular matrix (ECM) performs a crucial role in fostering anoikis resistance [33, 34]. Our bioinformatics analysis revealed a correlation between ARGs and ECM. In small cell lung cancer research, inhibition of the PI3K/AKT/mTOR pathway overrided chemotherapy resistance mediated by ECM [35]. Thus, we conjecture that the PI3K/AKT/mTOR signaling pathway significantly contributes to interactions between anoikis and ECM, however, further experimental validation is imperative. In conclusion, as a secreted protein, SPP1 acts on HCC cells via autocrine pathways and enhances resistance to anoikis by activating the PI3K/AKT/mTOR signaling pathway through integrin-mediated PKCα phosphorylation.

In the evolving landscape of HCC metastasis, the immune response assumes an obstacle that must not be underestimated. As a highly immunogenic tumor, HCC frequently exhibits a widespread infiltration by various immune cells in its surrounding area. Through an immunocomparative analysis of different clusters associated with ARGs, we observed that clusters with elevated expression levels of ARGs demonstrated an enhanced infiltration by diverse immune cells. It is widely believed that a “hot” tumor state is more conducive to immunotherapy [36]. However, our findings suggested that clusters with high expression levels of ARGs also exhibited a profusion of MDSCs and Tregs, which could explain why a “hot” tumor state actually led to a poorer prognosis. MDSCs, a heterogeneous group of immune cells derived from the bone marrow stem cell lineage, possess potent immunosuppressive functions [37]. Tregs in the tumor microenvironment facilitate tumor progression by curtailing anti-tumor immune responses [38]. Importantly, among the core target genes involved in ARGs, we identified that only SPP1 exhibited a significant association and positive correlation with MDSCs and Tregs. This further confirms SPP1 as a crucial gene in anoikis in HCC. Circulating tumor cells serve as the seeds for distant metastasis, necessitating exceptional resistance to anoikis and the ability to elude immune surveillance. In cancer patients, an escalation in the quantity of MDSCs and Tregs detected in circulation was correlated with an upsurge in the count of circulating tumor cells. Moreover, MDSCs presented in the portal vein blood of pancreatic cancer patients, clustered with circulating tumor cell and surrounding cells, aiding the proliferation and migration of circulating tumor cells in vitro [39, 40]. Elevated levels of circulating tumor cells and peripheral Tregs were prognostic of postoperative HCC recurrence [41]. However, the mechanisms governing these phenotypes remain unexplored. Previous studies have demonstrated that prostate tumor cells enlisted MDSCs through the release of SPP1 protein for migration [17], while scirrhous hepatocellular carcinoma cells inhibited dendritic cell function and hindered T-cell activation via the SPP1-CD44 axis [42]. Our research has revealed that SPP1 could beckon MDSCs and Tregs for migration purposes and animal experiments have substantiated a reduction in infiltrating MDSCs after downregulating SPP1 expression, offering robust evidence supporting our bioinformatics results and proposing potential mechanisms for the aforementioned clinical phenotypes. Furthermore, our study also discovered that under the induction of SPP1, pro-cancer genes such as Arg1 and Nos2 were upregulated in MDSCs, while anti-cancer genes like IL1b and TNFα were downregulated. This appeared to be contrary to the immune checkpoint function of MDSCs [43], further confirming the potential impact of SPP1 on MDSCs. Additionally, the expressions of numerous characteristic genes in Tregs were upregulated [44], confirming the role of SPP1 in enhancing the immunosuppressive function of Tregs. Therefore, we proposed that circulating hepatocellular carcinoma cells, by secreting SPP1, recruited MDSCs and Tregs and promoted their function to achieve immune escape.

Although our research has furnished novel insights and potential therapeutic targets for resisting anoikis and metastasis in HCC, certain limitations must be recognized. Firstly, although our bioinformatics analysis illuminated the involvement of multiple signaling pathways in the resistance to anoikis process, we did not delve into potential signaling pathways beyond the PI3K/AKT/mTOR axis, a well-established and highly ranked pathway. Secondly, our investigation of immune cells was not comprehensive, as we did not differentiate MDSCs into subtypes. It is crucial to note that both mMDSCs and pMNMDSCs exhibit immunosuppressive effects, but their roles vary significantly among different tumors [45–47]. Lastly, although we observed an association between SPP1 and MDSCs, as well as Tregs, we did not further explore the specific mechanisms of action of SPP1.

To sum up, the downregulation of SPP1 significantly reduced HCC cells’ resistance to anoikis, inhibited HCC growth and metastasis in vivo. Although other unknown mechanisms might contribute to the observations in this study, it is crucial to note that SPP1’s ability to confer resistance to anoikis serves as a crucial protective mechanism for HCC cells. This study analyzed bioinformatics data and experimentally validated SPP1 as a pivotal gene involved in HCC’s resistance to anoikis, unraveling an undocumented mechanism involving the promotion of anoikis resistance through the PI3K/AKT/mTOR signaling pathway. Furthermore, by recruiting cell migration and enhancing the function of immunosuppressive cells, including MDSCs and Tregs, SPP1 provides novel insights into its pathophysiological role in HCC metastasis and hinted at its potential as a therapeutic target for treating HCC.

## Electronic supplementary material

Below is the link to the electronic supplementary material.

Supplementary Material 1

Supplementary Material 2

Supplementary Material 3

## Data Availability

No datasets were generated or analysed during the current study.

## Abbreviations

ARGs: Anoikis-related genes
HLA: Human leukocyte antigen
ICI: Immune checkpoint inhibitor
TME: Tumor microenvironment
ECM: Extracellular matrix
