# Supplementary material for: The resistance to anoikis, mediated by Spp1, and the evasion of immune surveillance facilitate the invasion and metastasis of hepatocellular carcinoma
Source: Apoptosis. 2024 Jul 27;29(9-10):1564–83. doi: 10.1007/s10495-024-01994-x (PMC11416391; doi:10.1007/s10495-024-01994-x)
Supplement: Supplementary file 3 — Supplementary Material 3 [file 10495_2024_1994_MOESM3_ESM.pdf]

The resistance to anoikis, mediated by Spp1, and the evasion of immune surveillance facilitate the invasion and metastasis of hepatocellular carcinoma Apoptosis

Zhengwei Zhang [1†], Xiaoning Chen [1†], Yapeng Li [1†], Feng Zhang [2†], Zhen Quan [1], Zhuo Wang [2], Yang Yang [2], Wei Si [2], Yuting Xiong [2], Yu Bian [2*], Jiaming Ju [2*], Shibo Sun [1*]

[1]The Second Affiliated Hospital of Harbin Medical University, Harbin 150081, China

[2]Department of Pharmacology (National Key Laboratory of Frigid Zone Cardiovascular Diseases, the State-Province Key Laboratories of Biomedicine-Pharmaceutics of China, Key Laboratory of Cardiovascular Research, Ministry of Education), College of Pharmacy, Harbin Medical University, Harbin 150081, China

*** Correspondence:**

Shibo Sun, shibosun8@hrbmu.edu.cn； +86 0451 8660 5559;

Jiaming Ju, sonwjuhmu@qq.com;

Yu Bian, bianyu@hrbmu.edu.cn.

Supplementary Figure S1

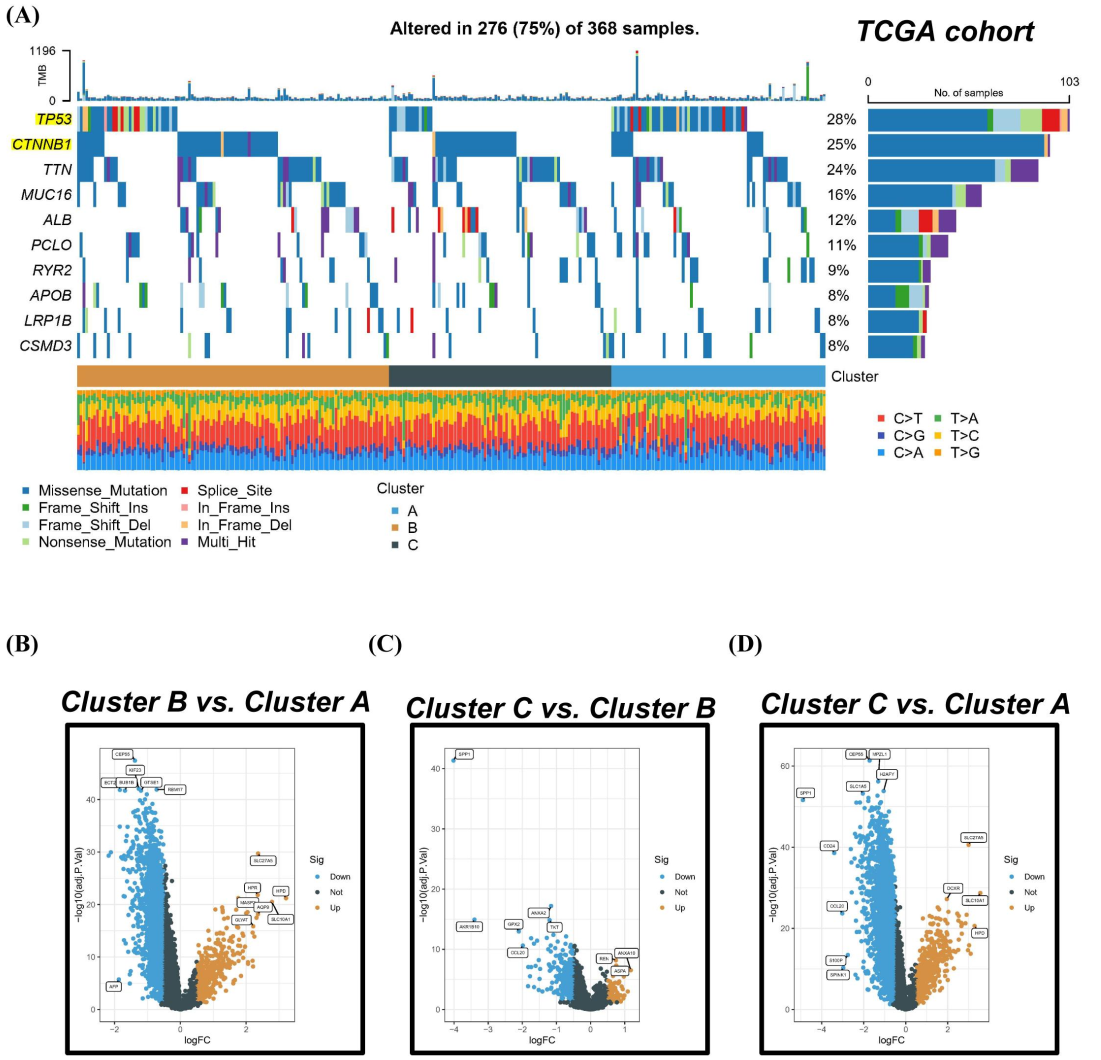

Identification and further analysis of prognostic ARGs clusters. (A) The mutation profiles of various subtypes were examined in order to investigate the factors contributing to disparate prognoses. (B, C and D) Common DEGs among different subtypes were identified.

Supplementary Figure S2

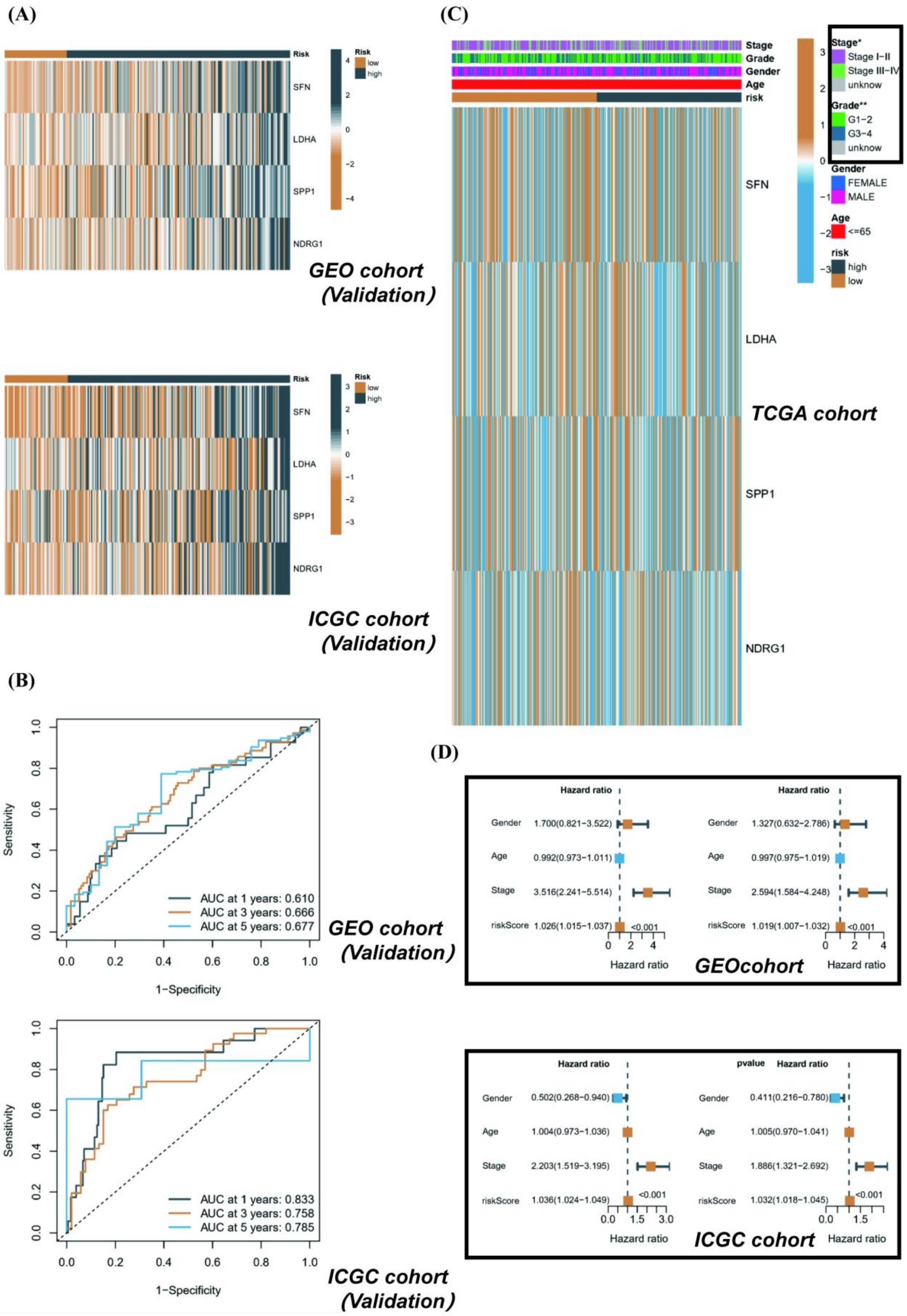

SFN, LDHA, SPP1 and NDRG1 are the core anoikis related prognostic genes in HCC. (A) The expression levels of SFN, LDHA, SPP1, and NDRG1 genes were investigated in the RSF model based on data from GEO and ICGC cohorts. (B) ROC curve analysis was performed on the risk prognostic model based on data from GEO

and ICGC cohorts. (C) Based on TCGA cohort, the differences in clinical characteristics between high and low risk groups were compared. (D) Univariate and multivariate Cox regression analyses were performed on risk score based on GEO and ICGC cohorts.

Supplementary Figure S3

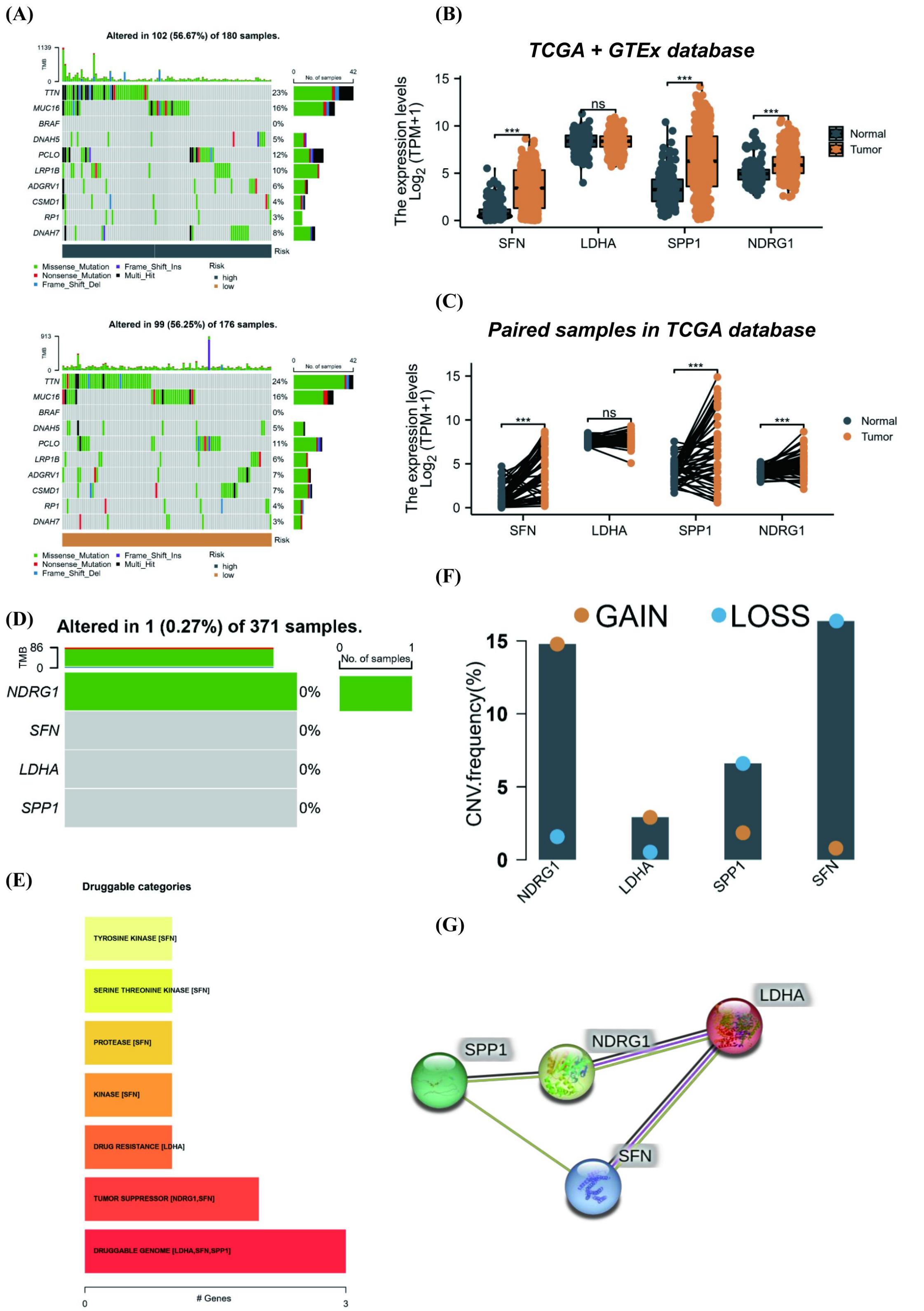

Comparative analysis of SFN, LDHA, SPP1 and NDRG1. (A) The mutated genes of patients with high and low risk were compared. (B and C) The expressions of SFN, LDHA, SPP1 and NDRG1 in different samples were compared. (D) The mutation

statuses of SFN, LDHA, SPP1 and NDRG1 were compared based on the TCGA-LIHC cohort. (E) The potential drug targets involved in SFN, LDHA, SPP1 and NDRG1 were analyzed. (F) CNVs of SFN, LDHA, SPP1, and NDRG1 were analyzed. (G) PPI network analysis was performed for SFN, LDHA, SPP1, and NDRG1. ***$p < 0.001$.

Supplementary Figure S4

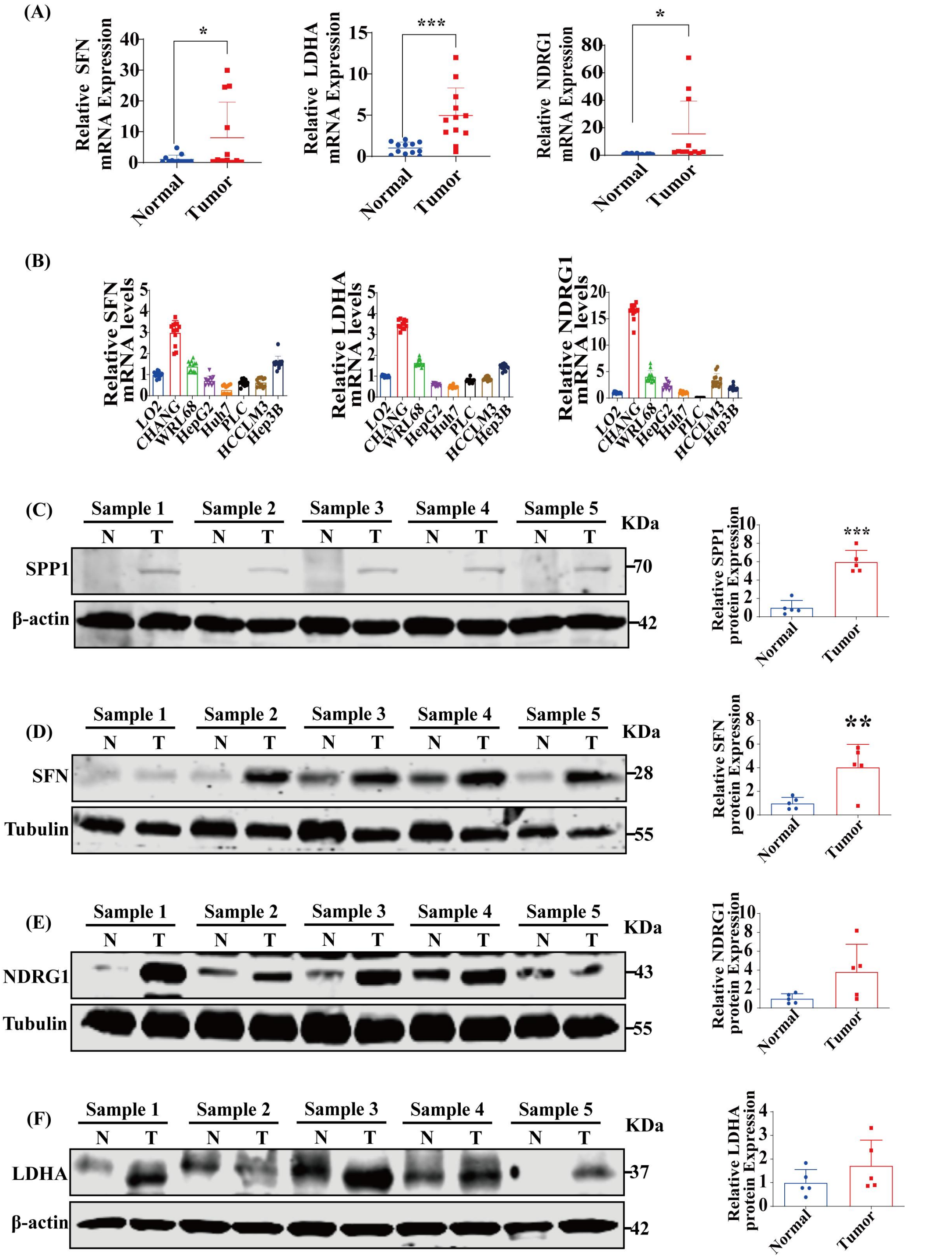

Expression analysis of SPP1, SFN, LDHA, and NDRG1. (A) The mRNA levels of SFN, LDHA, and NDRG1 in HCC and non-cancerous tissues were compared (n = 12). (B) The expression levels of SFN, LDHA, and NDRG1 mRNA in different HCC cell lines and normal liver cell lines were compared (n = 12). (C, D, E and F) The protein expression levels of SPP1, SFN, NDRG1 and LDHA in HCC and non-cancerous tissues were compared (n = 5). *$p < 0.05$, **$p < 0.01$, and ***$p < 0.001$.

Supplementary Figure 5

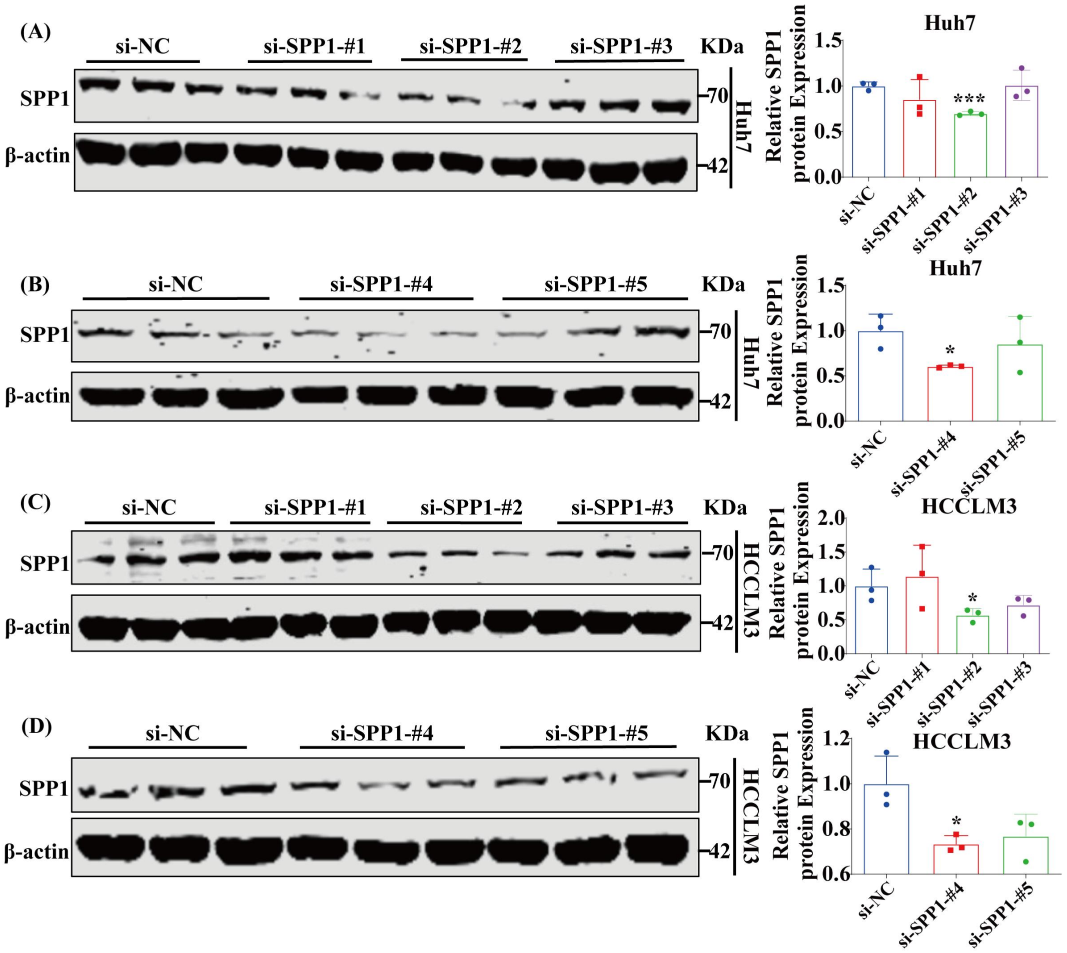

Validation of SPP1 knockdown efficiency. (A, B, C and D) The efficiency of SPP1 knockdown was verified in Huh7 and HCCLM3 cells (n = 3). *p < 0.05 and ***p < 0.001.

Supplementary Figure S6

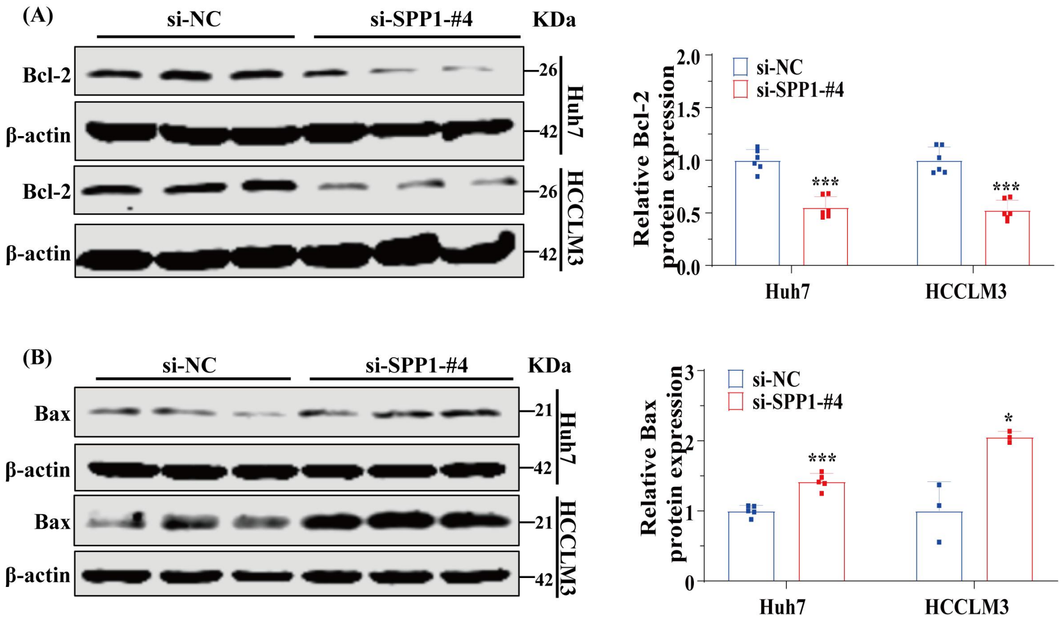

The SPP1 protein enhances anoikis resistance. (A and B) After SPP1-knockdown Huh7 and HCCLM3 cells were cultured in suspension for 48 h, the expressions of Bcl-2 and Bax were compared by Western blot (n = 3-6). *$p < 0.05$ and ***$p < 0.001$.

Supplementary Figure S7

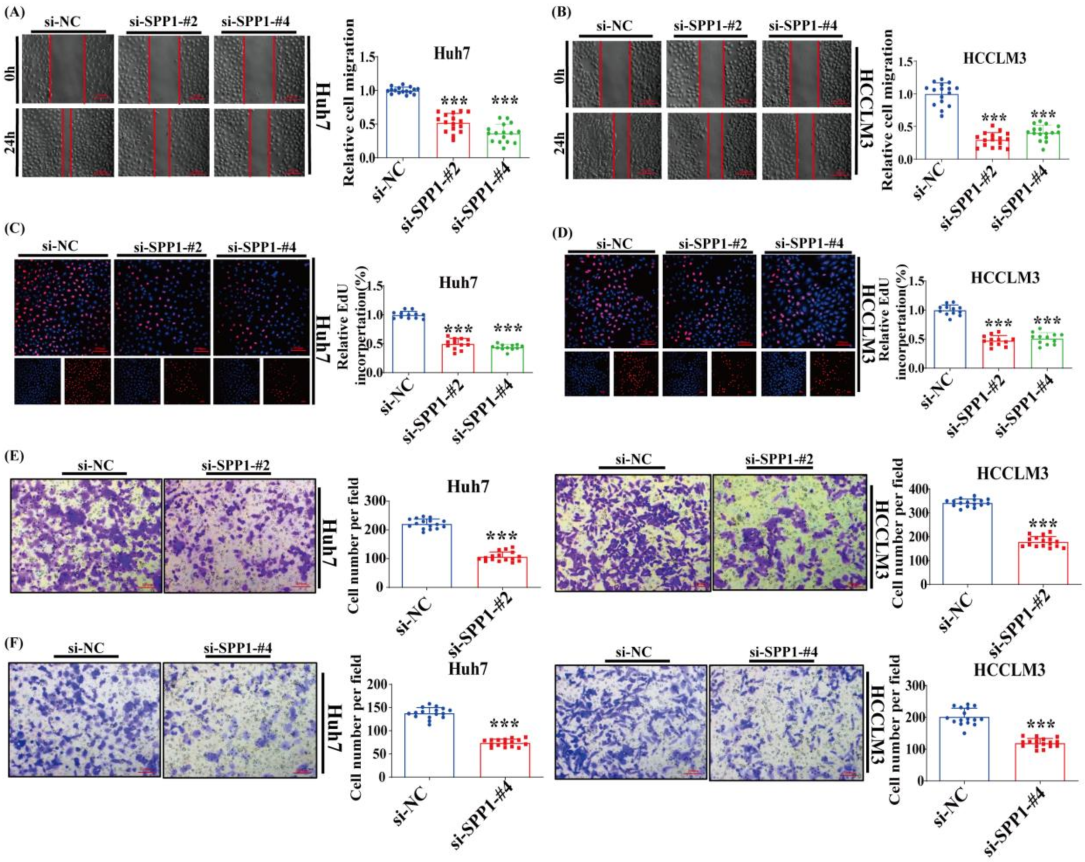

The SPP1 protein enhances malignant behaviors of HCC cells. (A and B) The wound healing assay was employed to assess the migratory capacity of Huh7 and HCCLM3 cells with SPP1-knockdown (n = 4). (C and D) The EDU assay was employed to assess the impact of SPP1-knockdown on the proliferation of Huh7 and HCCLM3 cells (n = 3). (E and F) The migration abilities of Huh7 and HCCLM3 cells were determined by transwell assays in SPP1- knockdown (n = 4). ***$p < 0.001$.

Supplementary Figure S8

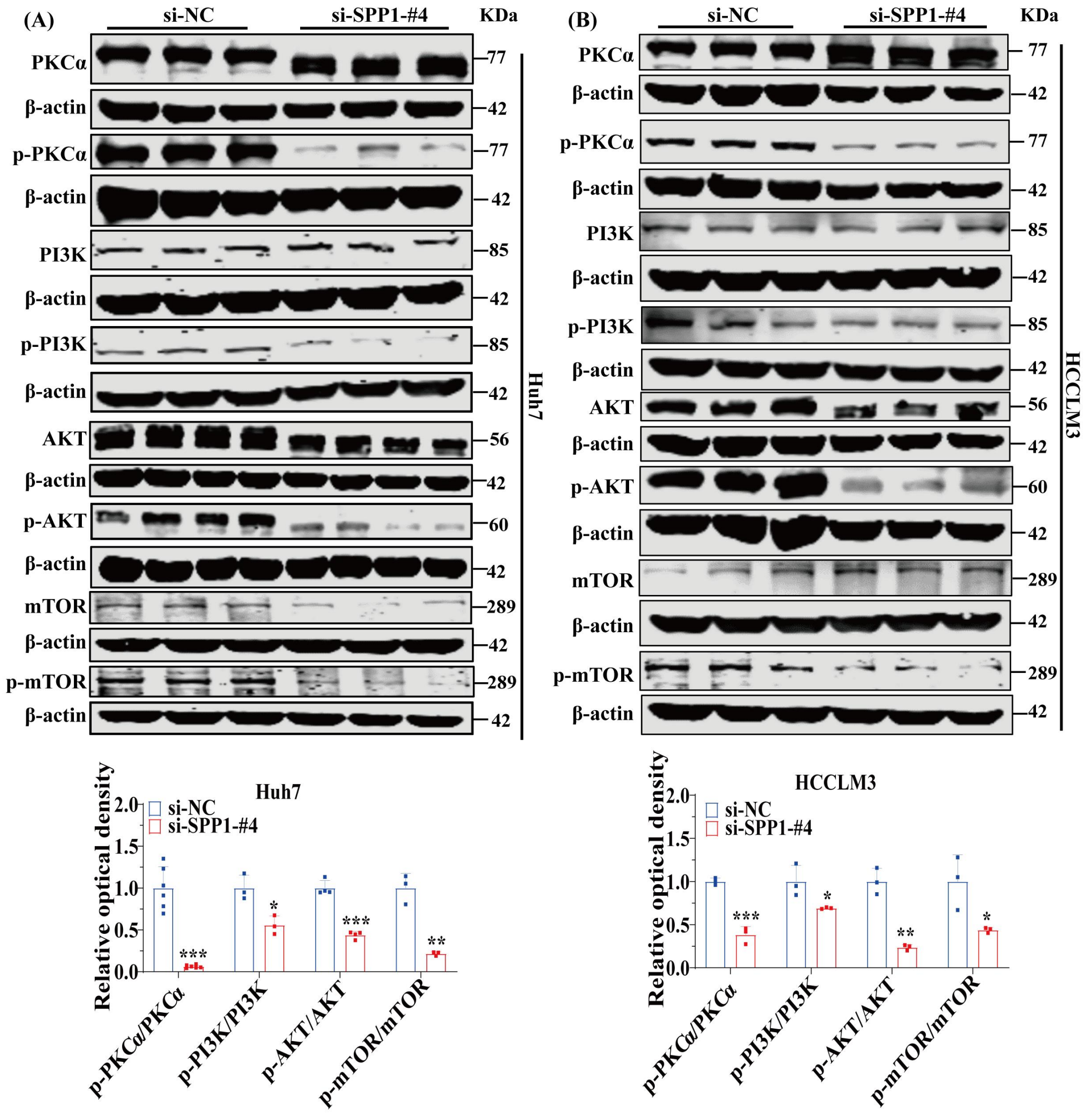

The SPP1 protein activates the PI3K/AKT/mTOR signaling pathway through PKCα phosphorylation to resist anoikis in HCC. (A and B) After SPP1-knockdown Huh7 and HCCLM3 cells were cultured in suspension for 48 h, the expressions of phospho-PKC, phospho-PI3K, phospho-AKT and phospho-mTOR were compared by Western blot (n = 3-6). *$p < 0.05$, **$p < 0.01$, and ***$p < 0.001$.

Supplementary Figure S9

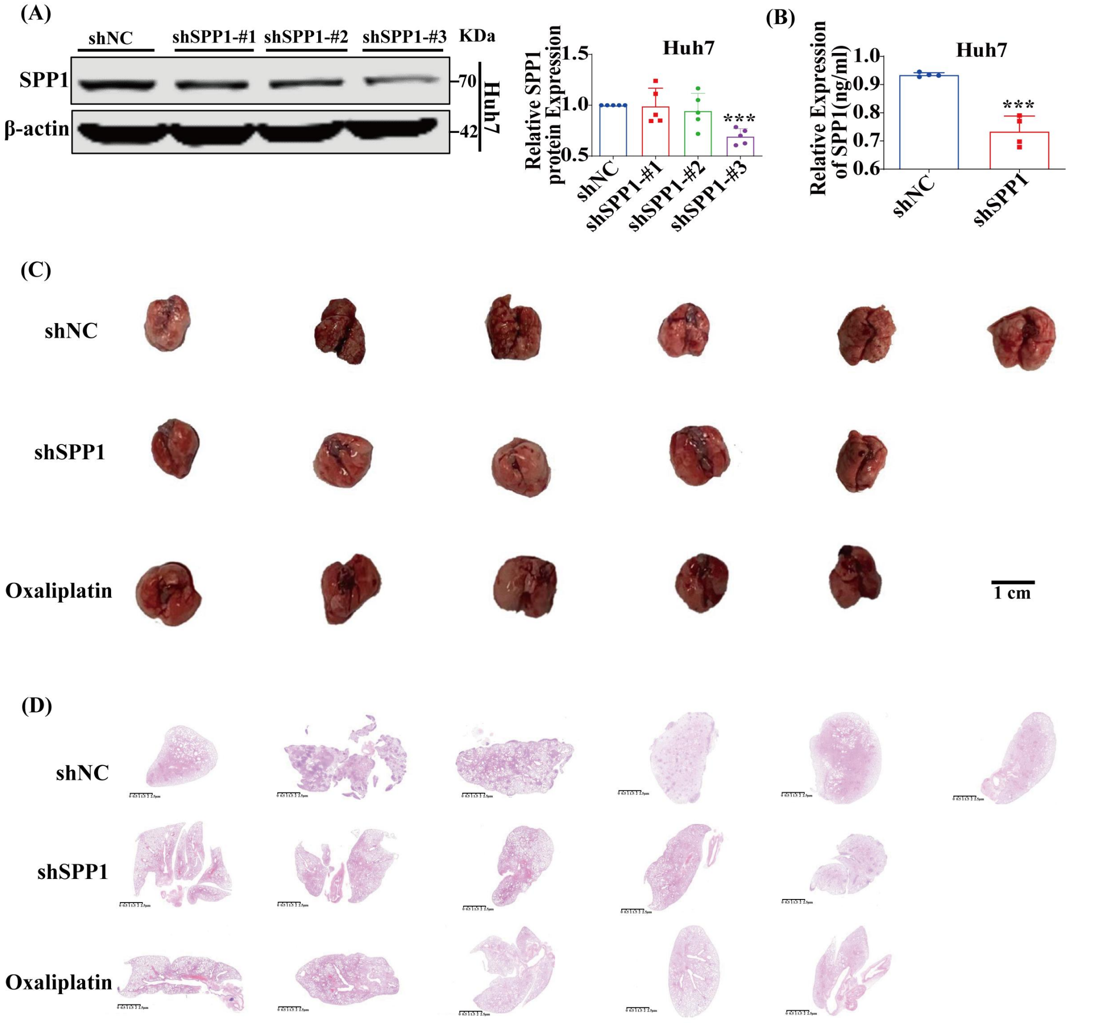

Downregulation of SPP1 suppresses HCC metastasis in vivo. (A) The efficiency of SPP1 knockdown was verified in Huh7 (n = 5). (B) ELISA kit was used to detect the concentration of SPP1 protein in Huh7 culture medium supernatant after SPP1-knockdown (n = 4). (C) Images of lung metastases were shown (n = 5-6). (D) H&E stained images of lungs were obtained from the shNC, shSPP1, and oxaliplatin groups. The scale bar in H&E stained tissue images was 2500 μm (n = 5-6). ***p < 0.001.
